# Supplementary material for: Engineering the ADDobody protein scaffold for generation of high-avidity ADDomer super-binders
Source: Structure. Author manuscript; Available in PMC 2024 Nov 15. (PMC7616808; doi:10.1016/j.str.2023.12.010)
Supplement: Supplemental information [file EMS199899-supplement-Supplemental_information.zip › 1-s2.0-S0969212623004513-mmc2.pdf]

Structure

Article

Engineering the ADDobody protein scaffold for generation of high-avidity ADDomer super-binders

Graphical abstract

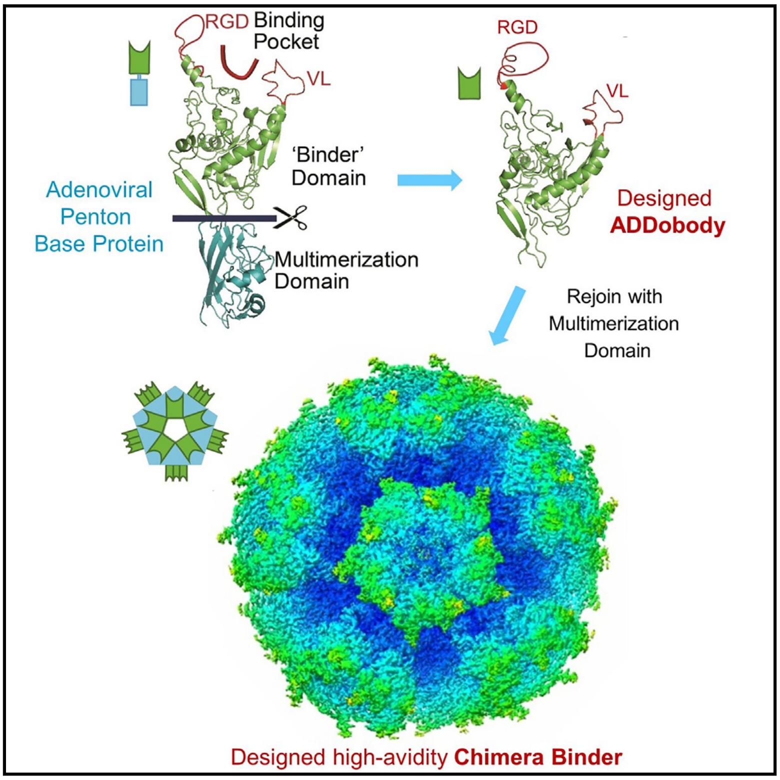

Highlights

- Design of ADDobody with high expression yields, thermostability, and loop variability
- Crystal structures of ADDobody validate design
- 10^4-fold enrichment of binders over non-binders by ADDobody ribosome display selections
- ADDobody conversion into nanoparticles with 60 binding sites and ultra-high avidity

Authors

Dora Buzas, Huan Sun, Christine Toelzer, ..., Frederic Garzoni, Imre Berger, Christiane Schaffitzel

Correspondence

imre.berger@bristol.ac.uk (I.B.), cb14941@bristol.ac.uk (C.S.)

In brief

Based on an adenoviral protein, Buzas et al. engineer a novel protein scaffold, the ADDobody, validated by high-resolution structure analyses. ADDobody can be functionalized to interact with a chosen antigen. Fusing ADDobody to a multimerization domain results in a megadalton-sized protein nanoparticle binding the antigen extremely tightly due to avidity.

Buzas et al., 2024, Structure *32*, 342–351
March 7, 2024 © 2023 The Author(s). Published by Elsevier Ltd.
https://doi.org/10.1016/j.str.2023.12.010

CellPress

CellPress
OPEN ACCESS

Structure

Article

Engineering the ADDobody protein scaffold for generation of high-avidity ADDomer super-binders

Dora Buzas,[1,2,6] Huan Sun,[1,2,5,6] Christine Toelzer,[1] Sathish K.N. Yadav,[1] Ufuk Borucu,[1] Gunjan Gautam,[1] Kapil Gupta,[1,3] Joshua C. Bufton,[1] Julien Capin,[1] Richard B. Sessions,[1] Frederic Garzoni,[3] Imre Berger,[1,2,4,*] and Christiane Schaffitzel[1,7,*]

[1]School of Biochemistry, University of Bristol, University Walk, Bristol BS8 1TD, UK
[2]Max Planck Bristol Centre for Minimal Biology, Cantock's Close, Bristol BS8 1TS, UK
[3]Imophoron Ltd, Science Creates Old Market, Midland Road, Bristol BS2 0JZ, UK
[4]School of Chemistry, University of Bristol, Cantock's Close, Bristol BS8 1TS, UK
[5]Present address: The Institute of Medicinal Plant Development (IMPLAD), No. 151 Malianwa North Road, Haidian District, Beijing 100193, P.R. China
[6]These authors contributed equally
[7]Lead contact
*Correspondence: imre.berger@bristol.ac.uk (I.B.), cb14941@bristol.ac.uk (C.S.)
https://doi.org/10.1016/j.str.2023.12.010

SUMMARY

Adenovirus-derived nanoparticles (ADDomer) comprise 60 copies of adenovirus penton base protein (PBP). ADDomer is thermostable, rendering the storage, transport, and deployment of ADDomer-based therapeutics independent of a cold chain. To expand the scope of ADDomers for new applications, we engineered ADDobodies, representing PBP crown domain, genetically separated from PBP multimerization domain. We inserted heterologous sequences into hyper-variable loops, resulting in monomeric, thermostable ADDobodies expressed at high yields in *Escherichia coli.* The X-ray structure of an ADDobody prototype validated our design. ADDobodies can be used in ribosome display experiments to select a specific binder against a target, with an enrichment factor of $\sim10^4$-fold per round. ADDobodies can be re-converted into ADDomers by genetically reconnecting the selected ADDobody with the PBP multimerization domain from a different species, giving rise to a multivalent nanoparticle, called Chimera, confirmed by a 2.2 Å electron cryo-microscopy structure. Chimera comprises 60 binding sites, resulting in ultra-high, picomolar avidity to the target.

INTRODUCTION

High-specificity and high-affinity monoclonal antibodies are vital reagents in modern biomedicine and biotechnology. Phage display and yeast display are commonly used technologies to select antibodies using libraries from immunized animals.[1,2] Nonetheless, both display technologies are limited in the size of the antibody library due to necessary transformation and amplification steps in cells. The ribosome display *in vitro* selection technology avoids any *in vivo* steps that limit the diversity of the library and thus allows selecting binders from very large libraries (up to $\sim10^{12}$). Therefore, ribosome display is particularly suited for naive and synthetic libraries with a lower content of potential binders (in contrast to antibody libraries generated from immunized animals).[3,4] Moreover, ribosome display allows the selection and evolution of antibodies *in vitro* under user-defined selection conditions, independent of the immunogenicity or toxicity of the antigen, also against highly conserved targets.[5,6] Importantly, affinities in the picomolar and femtomolar range have been reported for *in vitro* selected antibodies, far exceeding the affinities found in natural antibodies.[7,8]

Alternative binder scaffolds are based on non-immunoglobulin domains and ideally have the following characteristics: improved biophysical properties, small size and binding of their target with similar or higher affinity, and/or specificity as compared to antibodies. Furthermore, alternative binder scaffolds can be produced with high yields as recombinant proteins in *Escherichia coli* (low cost).[9] In order to avoid misfolding, optimize yields, and permit expression in the reducing environment of the cytoplasm, disulfide bonds or cysteines are usually avoided in alternative binder scaffolds. Alternative binders have many applications including purification, detection and quantification of their target proteins, facilitating structure elucidation, live imaging, for targeted drug delivery, and as protein-based therapeutic.[9–15]

We previously developed ADDomer (adenovirus-derived dodecamer) as a thermostable scaffold with low intrinsic

This is an open access article under the CC BY license (http://creativecommons.org/licenses/by/4.0/).

CellPress
OPEN ACCESS

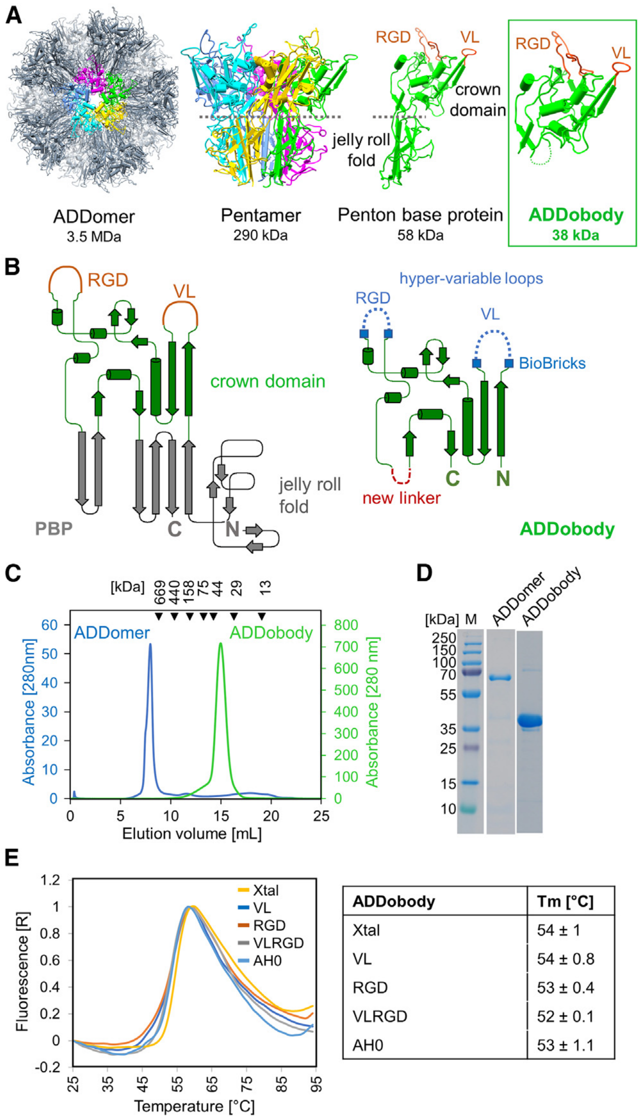

ADDobody	Tm [°C]
Xtal	54 ± 1
VL	54 ± 0.8
RGD	53 ± 0.4
VLRGD	52 ± 0.1
AH0	53 ± 1.1

Figure 1. ADDobody protein design

(A) ADDomer (left, PDB: 6HCR[16]) assembles spontaneously from twelve pentons (middle left) derived from the adenovirus penton base protein (PBP) (middle right). Each PBP comprises a jelly-roll multimerization domain and a head domain (crown) with flexible VL and RGD loop regions (orange). ADDobody (right) consists of the isolated, engineered crown domain.

(B) Topology drawing for the PBP (left) as observed in ADDomer and the designed ADDobody (right). In the BioBrick format, the transition between the protein scaffold and the hyper-variable loop regions (blue) is marked by restriction site sequences (blue boxes).

(C) Size-exclusion chromatography profile of ADDomer (in the void volume) and monomeric ADDobody which elutes at the expected volume for the size of a ~38 kDa protein using a Superdex 200 10/300 GL column.

(D) Coomassie-stained SDS-PAGE showing purified ADDomer/PBP and ADDobody. Left: Molecular weight marker.

(E) Thermal unfolding curves of different ADDobody constructs and resulting melting temperatures (Tm) (n = 3) are shown for ADDobodies used in this study.

immunogenicity and demonstrated its use as a novel vaccine nanoparticle.[16,17] ADDomer is based on the penton base protein (PBP) of human adenovirus Ad3 serotype and exhibits intrinsic self-organizational properties in the test tube. In the ADDomer, the PBP monomers spontaneously assembles to form a pentamer, and twelve pentamers organize into a dodecamer (Figure 1A) characterized by high stability and thermotolerance exceeding 50°C.[16–18] The PBP monomer folds into two distinctive domains (Figures 1A and 1B); the jelly-roll fold domain which is located in the core of the dodecamer and assumed to be responsible for multimerization, and a solvent-facing exterior domain, called the crown domain.[16] The crown domain comprises two highly flexible regions, called variable loop (VL) and arginine-glycine-aspartic acid (RGD) motif-containing loop (Figure 1A). These flexible loop regions were identified as versatile insertion sites that can be used for displaying foreign sequences such as immunogenic epitopes for genetically encoded multiepitope display.[16,17,19] The display of multiple pathogen-derived peptide epitopes on the surface of ADDomer enabled the design of vaccine candidates which could elicit strong specific immunoglobulin responses against Chikungunya virus,[16] severe acute respiratory syndrome coronavirus 2 (SARS-CoV-2) virus,[20] and foot-and-mouth disease.[21] Importantly, the high thermostability (>50°C) of ADDomer enables refrigeration-independent distribution and storage.[16] Also, lyophilization of ADDomers is possible.[22] ADDomer-based thermostable vaccines or therapeutics, due to thermostability, could be deployed independent of a cold chain, which is a highly significant criterium given that each year ~50% of vaccine dose wastage is due to cold chain disruptions worldwide.[23]

Here, we designed ADDobody, a new scaffold protein based on the crown domain of the PBP (Figures 1A and 1B). We show that ADDobodies exhibit the advantages of alternative protein scaffolds outlined previously: ADDobodies are single-domain, monomeric proteins with comparatively small molecular weight (~38 kDa), exhibit a highly stable structure without any disulfide bonds, and can be produced at high yields using *E. coli.* Of note, ADDobodies retain the thermostability of the ADDomer—our designed ADDobody prototype exhibits a melting temperature (Tm) of 54°C. We determined two crystal structures of ADDobody at 2.9 and 3.2 Å resolution, respectively, validating our design. Moreover, we show that ADDobody tolerates insertion of sequences of variable lengths into the hypervariable loops (VL and RGD), retaining high expression yields and melting temperature of the ADDobody prototype, which is a prerequisite for randomization of the loops to generate a future synthetic ADDobody library. In a proof-of-concept experiment, we demonstrate that ADDobody can be used for *in vitro* selection by ribosome display with an enrichment factor of ~10^4-fold per selection round.

By genetically fusing ADDobody and the multimerization jelly-roll fold domain, ADDobody can be converted back into a nanoparticle with 60 binding sites against the target protein, resulting in ultra-high avidity. Specifically, we rejoined ADDobody derived from human adenovirus Ad3 with a jelly-roll fold domain from a chimpanzee adenovirus, giving rise to a novel functional chimeric ADDomer nanoparticle, which we call "Chimera." We solve the structure of this Chimera nanoparticle by cryogenic electron microscopy (cryo-EM). Chimera binds its target with ultra-high (picomolar) avidity corroborating our concept that super-binders can be engineered which bind their target with high efficiency. We anticipate that such ADDomer nanoparticles

generated from selected ADDobody binders could be highly useful molecular tools for efficient detection, binding, and neutralization of diverse pathogenic or toxic targets.

RESULTS

Design and biophysical characterization of ADDobody constructs

We set out to design and produce the crown domain of human adenovirus Ad3 PBP in isolation, and to determine the biophysical and biochemical properties, and the structure of the resulting protein, which we call ADDobody. For this, the crown domain had to be separated from the jelly-roll fold domain, and the N- and C-terminal parts reconnected by introducing a linker (Figures 1A and 1B). To this end, two beta-strands comprising residues 423–456 were deleted that are part of the jelly-roll fold domain of the Ad3 PBP (Figure 1B).[16] We replaced this part by a flexible 6 amino acid residue linker with the sequence NGDSGN, reconnecting the crown polypeptide chain (Figure 1B). The VL and RGD loops of the PBP are hyper-variable in length and sequence in nature[24] and, as we have shown previously for ADDomer, represent versatile insertion sites for heterologous amino acid sequences seemingly not constrained by length and sequence context.[16] Therefore, we reasoned that randomized sequences could be introduced also in the VL and RGD loops of ADDobody, facilitating the future generation of a library comprising randomized loops. We chose a “BioBrick” format[25] to delineate the boundaries of backbone and loops in the ADDobody scaffold. The position of the restriction sites was based on the ADDomer structure (PDB: 6HCR)[16] (Figure 1B).

To validate our design by high-resolution structural analysis using X-ray crystallography, we created an ADDobody prototype with minimized loops, called ADDobody-Xtal, based on the assumption that long flexible loops could be detrimental to crystal lattice formation. Moreover, we designed exemplar ADDobodies comprising extended loop sequences in the VL loop (ADDobody-VL), in the RGD loop (ADDobody-RGD), in both VL and RGD loops (ADDobody-VLRGD) in BioBrick format. Finally, we designed an ADDobody comprising a viral epitope sequence, AH0, inserted into VL (ADDobody-AH0). The AH0 sequence is part of the receptor-binding motif (residues Y473 to Y505) of the ancestral strain SARS-CoV-2 spike protein,[17] which mediates binding to the human host cell receptor angiotensin-converting enzyme 2.[26] We have shown previously that the AH0 sequence is recognized and tightly bound by a camelid VHH nanobody, ADAH11.[17] The amino acid sequences of the ADDobodies used in this study are provided in Figure S1.

All ADDobody constructs were successfully expressed in *E. coli* BL21(DE3) with high yields (>10 mg/L cell culture) within 3 h at 30°C. ADDobodies were affinity purified via the His-tag, followed by anion exchange and size-exclusion chromatography (SEC) (Figures 1C, 1D, and S2). ADDobodies eluted at ~15 mL from a Superdex200 column as expected for a monomer with a molecular weight of ~38 kDa (Figures 1C and 1D). For comparison, the PBP (~64 kDa) forms a dodecamer of pentons (the ADDomer) with a size of 3.4 MDa and elutes close to the void volume (~7 mL) (Figure 1C).

To determine the stability of our newly designed proteins, purified ADDobodies were analyzed by thermal unfolding using a Thermofluor assay.[27] The Tm for all ADDobody constructs, ranged from 52°C to 54°C (Figure 1E) indicating that the ADDobody scaffold adopts a robust fold, irrespective of the individual loop lengths and sequences or the introduction of the linker, none of which seemingly affected thermostability. For comparison, the ADDomer nanoparticle exhibits a Tm of 54°C.[16] In summary, we successfully designed an ADDobody scaffold that has excellent expression yields in *E. coli*, maintains thermostability, and tolerates diverse amino acid sequence contexts and lengths in its loops, recapitulating favorable characteristics of the ADDomer nanoparticle from which ADDobody is derived.

Crystal structures of ADDobody

ADDobody with minimized VL and RGD loop sizes (ADDobody-Xtal, Figure S1) was crystallized. Crystals grew under two different conditions, one containing 0.1 M zinc acetate and the other devoid of zinc. During X-ray diffraction data collection and structure determination (Table 1), two different crystal forms were observed: In the absence of zinc ions, ADDobody crystals adopted space group P1 and diffracted to a resolution of 3.17 Å. ADDobody crystals grown in the presence of zinc adopted space group $P2_12_12_1$, and diffracted to a resolution of 2.90 Å. The ADDobody structures from both crystal forms show identical scaffolds while the VL and RGD loops appear flexible (Figures 2A and S3). Superimposition of ADDobodies from the crystals with the crown domain from the ADDomer cryo-EM structure revealed a virtually identical fold with a root-mean-square deviation (RMSD) of 1.12 Å (Figure 2B). Three segments in the ADDobody crystal structures were partially unresolved to varying degrees in the individual chains in the asymmetric units, including VL and RGD loops, and a region close to the C-terminus comprising residues 270–305 (Figure S1). In the zinc-free ADDobody crystal form, the VL loop could be manually traced through the density in some of the monomers (Figure S3). The RGD loop region clearly lacked density for about 12–15 residues in all chains of both refined structures indicating conformational disorder (Figures S3 and 2B).

In the zinc-free ADDobody crystal form, twenty ADDobody monomers are found in the asymmetric unit (Figure 2C). Of note, five ADDobody monomers formed a penton in the crystal reminiscent of the ring-like arrangement in pentons formed by the PBP in the ADDomer (Figures 2C and 2D). Two such penton rings in the zinc-free triclinic crystal form a barrel-like decamer where the VL and RGD loops are outward facing and accessible (Figures 2C and 2D). The Coulomb potential indicates a complementary patch of negative and positive charges at the ADDobody surface which contribute to penton ring formation in the crystals (Figures S4 and 2E). Specifically, D106 forms a charge-charge interaction with R83. E13 and E58 are both in close proximity to R237 (Figure 2E). The stacking of the two penton rings is mostly mediated by van der Waals interactions, with residue F153 playing a central role (Figure 2F). A cation-Π interaction is formed between F153 and R140 of an adjacent chain, and F16 could also interact with F153 of the neighboring ADDobody polypeptide chain (Figure 2F). The crystallization of ADDobody proteins in the presence of zinc resulted in crystals with a characteristic needle-like shape. In this zinc-containing crystal form, ADDobodies also adopted a pentamer (Figure 2G); however, the pentameric rings are not stacked on top of each other.

CellPress
OPEN ACCESS

Table 1. ADDobody crystallographic data collection and refinement statistics

Data collection		
Structure	**zinc-free ADDobody**	**zinc-containing ADDobody**
PDB code	8COI	8QB3
Wavelength (Å)	0.9795	0.9780
Synchrotron (Beamline)	Diamond Light Source (I03)	Diamond Light Source (I04)
**Cell dimension**		
Space group	P1	$P\ 2_1\ 2_1\ 2_1$
a, b, c (Å)	103.85 104.63 180.49	101.48 103.07 167.22
α, β, γ (º)	92.03 95.65 112.61	90.00 90.00 90.00
**Collection statistics**		
Resolution range (Å)	96.41–3.17 (3.28–3.17)[a]	87.74–2.90 (3.0–2.9)[a]
Completeness (%)	98.6 (98.7)[a]	99.9 (99.6)[a]
Rmerge	0.102 (0.687)[a]	0.423 (1.339)[a]
Rmeas	0.120 (0.815)[a]	0.440 (1.397)[a]
CC 1/2	0.997 (0.723)[a]	0.946 (0.603)[a]
Signal-to-noise ratio (I/σ)	8.88 (1.55)[a]	2.35 (0.36)[a]
Total reflections	420813 (40470)[a]	504638 (47613)[a]
Unique reflections	116437 (11604)[a]	39590 (3875)[a]
Multiplicity	3.6 (3.5)[a]	12.7 (12.3)[a]
Wilson B-factor ($Å^2$)	83.61	26.72
**Refinement statistics**		
Rwork (%)	21.30	27.30
Rfree (%)	26.13	28.99
RMS bonds (Å)	0.002	0.003
RMS angles (°)	0.50	0.52
Ramachandran favored (%)	91.93	93.31
Ramachandran allowed (%)	7.89	6.59
Ramachandran outliers (%)	0.17	0.09
Rotamer outliers (%)	2.96	1.98
Average B-factor ($Å^2$)	91.32	31.97
Clash score	3.79	5.05
MolProbity score	2.01	1.81

[a]Highest resolution shell values in brackets.

Rather, a zinc ion mediates the interaction between pentamer rings participating in crystal packing. The zinc ion is coordinated by four residues from four different ADDobody chains: H151 donated from one chain, E13 from an adjacent ADDobody chain of the same pentamer, and H151 and E13 from the juxtaposed pentameric ring (Figures 2G and S5).

Taken together, the crystal structures validated our design, confirming that the crown domain adopts a stable fold with flexible loops. Moreover, the structures imply that multimerization on the level of the pentamer in the ADDomer nanoparticle may not be mediated by the jelly-roll fold domains alone, but that the crown domains also contribute, given that the ADDobodies were found to form pentameric rings in both crystal forms, in spite of being monomeric in solution (Figure 1C).

ADDobodies can be used for *in vitro* selection by ribosome display

For a proof-of-concept selection experiment, we cloned two ADDobody variants, ADDobody-AH0 and ADDobody-57. ADDobody-AH0 comprises a viral epitope, AH0, inserted in VL (Figure S1). AH0 interacts tightly with a specific target antigen, ADAH11.[17] ADDobody-57, in contrast, contains unrelated sequences in VL and RGD loops, and does not bind ADAH11. The constructs were converted into the ribosome display format by removing stop codons and adding a C-terminal spacer sequence derived from TonB fused in frame to the ADDobody-coding sequences.[28] In addition, a T7 promoter and a ribosome-binding site (Shine Dalgarno sequence) were introduced[28] (Figure 3A). Importantly, the AH0 encoding DNA sequence comprises a unique restriction site for restriction enzyme *Pst*I. Therefore, *Pst*I restriction digestion can be used to discriminate the PCR product encoding ADDobody-AH0 from the ADDobody-57 encoding construct which remains uncut (Figure 3A). In agarose gel electrophoresis, the PCR fragment representing uncut ADDobody runs at ∼1620 bp while the *Pst*I-digested AH0 construct gives rise to two bands at ∼1400 and 242 bp (Figure 3A).

Ribosome display *in vitro* selections were performed using biotinylated ADAH11 as the bait (Figures 3B and S6). When only the ADDobody-AH0 encoding construct was used for ribosome display selection, the recovered PCR product was completely digested by *Pst*I enzyme (Figures 3C and 3D). This confirms that the PCR product encodes ADDobody-AH0 and that ADDobody-AH0 binds its ADAH11 target. Furthermore, this experiment suggests that ADDobody-AH0 is properly displayed by the nascent mRNA-ribosome complexes tethered to the ADDobody due to the deleted stop codon, because the corresponding mRNA encoding ADDobody-AH0 could be efficiently recovered. To test the specificity of binding and the enrichment efficiency of ribosome display selections using ADDobodies, mRNA encoding ADDobody-AH0 was diluted in mRNA encoding ADDobody-57 at a ratio of 1:1, $1{:}10^3$, $1{:}10^5$, $1{:}10^7$, and $1{:}10^9$. These mRNA mixtures were then used for ribosome display selection experiments (Figure 3B). We asked whether ADDobody-AH0 could be recovered from this dilution based on ribosome display selection against its target antigen, ADAH11. After the first round of ribosome display selection, ADDobody-AH0 could be detected in the DNA pools of the 1:1 and $1{:}10^3$ dilutions (Figures 3C and S7) as evidenced by the presence of bands of ∼1,400 and 242 bp. In contrast, dilutions $1{:}10^5$, $1{:}10^7$, and $1{:}10^9$ resulted in PCR products that could not be digested by *Pst*I (Figures 3C and S7), indicating the prevalence of ADDobody-57. These PCR pools were subjected to a second round of ribosome display selection. The resulting PCR pools after round 2 were again *Pst*I digested. All experiments showed at least a partial digestion of the full-size product (bands at ∼1,600, ∼1,400, and 242 bp) evidencing co-existence of ADDobody-AH0 and

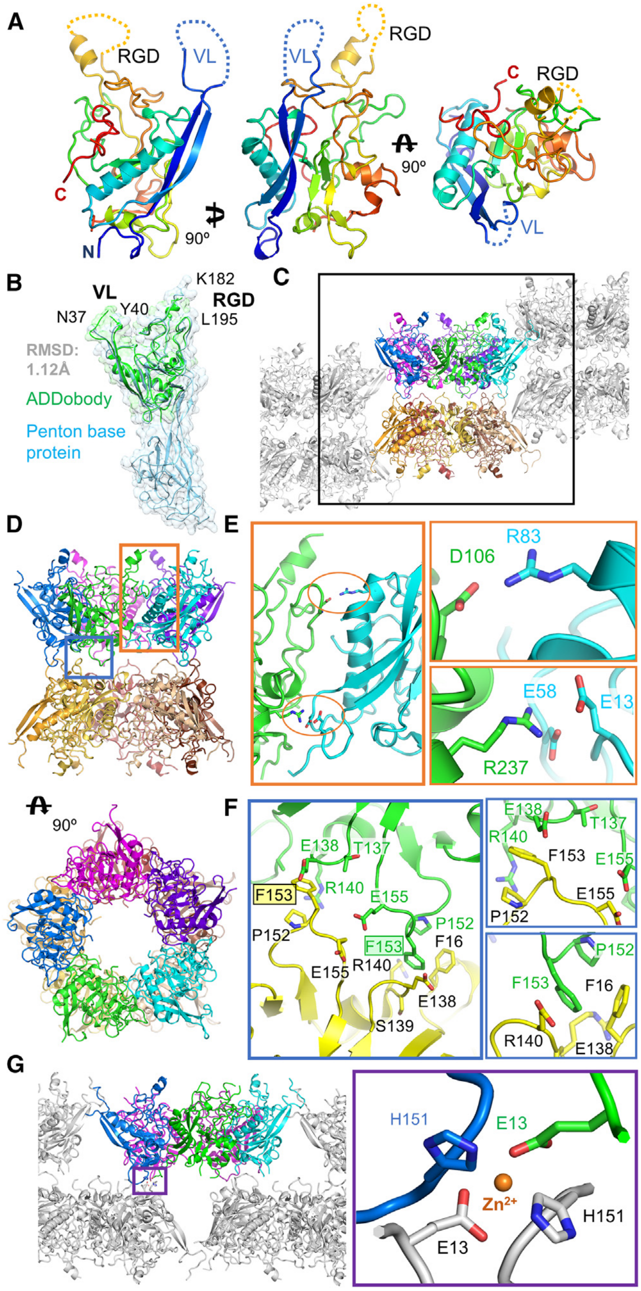

Figure 2. ADDobody crystal structures

(A) ADDobody structure in a front, side, and top view. The flexible RGD (yellow) and VL (blue) loops are indicated with dashed lines; the N-terminus (blue) and C-terminus (red) are marked.

(B) Alignment of ADDobody (green) and PBP (cyan, PDB: 6HCR[16]) crown domain with RMSD of 1.12 Å.

(C) Decameric organization observed in the ADDobody crystal. Two pentons stack together with flexible loop regions facing outwards. A side-view representation is shown with one decamer in color. Black box: unit cell.

(D) Side and top view of the ADDobody decamer forming a barrel.

(E) Left: Close-up view of the ADDobody interface within the penton (orange box in panel D). Right: charge-charge interactions stabilizing the interface.

(F) Left: Close-up view of the interface between ADDobody pentons in the decamer (blue box in panel D). Right: Hydrophobic contacts stabilizing the interface.

(G) Left: Zn^{2+}-containing crystal form showing ADDobody pentons (purple box highlights interface). Right: Zn^{2+} coordination by His151 and Glu13 residues promotes a crystal contact between two symmetry related pentons.

ADDobody-57 (Figures 3C, 3D, and S7). Based on these experiments with the 1:10^7 and 1:10^9 dilutions of AH0 (Figure 3D), we estimated an enrichment factor of ~10,000 for ADDobody-AH0 per ribosome display selection round against its cognate target antigen ADAH11.

To summarize, our ribosome display experiments demonstrate that ADDobody can be properly displayed on the ribosome suitable for *in vitro* selection. The mRNA-ribosome-ADDobody complexes can be used for selection against a cognate target, and ADDobodies binding to the target can be efficiently enriched over non-binding ADDobodies in a ribosome display selection experiment. This is a prerequisite for future selections of ADDobodies from a synthetic library with randomized VL and RGD loop sequences which can conceivably encompass up to 10^{12} members.[29]

Design and cryo-EM structure of Chimera super-binder

To test our concept that a selected ADDobody can be reconnected with a jelly-roll fold multimerization domain to yield ultra-high affinity ADDomer-based super-binders, we set out to graft ADDobody-AH0, derived from human adenovirus Ad3, onto the jelly-roll fold of the chimpanzee adenovirus serotype Y25 PBP (Figure 4A) to create a chimeric ADDomer, called Chimera, displaying 60 copies of AH0. This Chimera AH0 nanoparticle was produced using baculovirus/insect cell expression and purified by SEC and ion-exchange chromatography to homogeneity (Figure S8A). Chimera exhibits a Tm of 54°C in thermal shift experiments[27] (Figure S8B), virtually identical to the previously determined ADDomer Ad3 melting temperature.[16] We confirmed ADDobody-AH0 binding to ADAH11 by SEC and surface plasmon resonance (SPR) (Figures 4B and S9). In SEC, a peak was observed at ~14 mL with ADDobody-AH0 and ADAH11 co-eluting at the expected molecular weight (~50 kDa) (Figure S9), evidencing complex formation. A binding constant (K_D) of ~82 nM was determined by SPR (Figure 4B). Next, binding of Chimera AH0 to ADAH11 was analyzed. Chimera AH0 was passed at concentrations ranging from 0.325 to 1 nM over immobilized ADAH11, revealing strikingly low dissociation kinetics and an estimated avidity in the picomolar range (~200 pM, Figure 4C).

Finally, to validate our Chimera design at near-atomic resolution, we determined the structure by cryo-EM. Highly purified Chimera was used for data collection (Figure S10; Table S1). After two-dimensional and three-dimensional (3D) classification and 3D refinement with applying icosahedral symmetry, we obtained a 2.2 Å resolution structure from 377,978 particles (Figures 4A, S10, and S11), resulting in the highest resolution for any ADDomer-derived nanoparticle structure to date. Chimera displays the characteristic overall structure observed previously for the icosahedral ADDomer derived from human adenovirus Ad3 serotype, adopting a dodecamer of pentons (PDB: 6HCR).[16] The overlay of the ADDomer Ad3 jelly-roll fold and the chimpanzee jelly-roll fold domain in our cryo-EM structure reveal a virtually identical

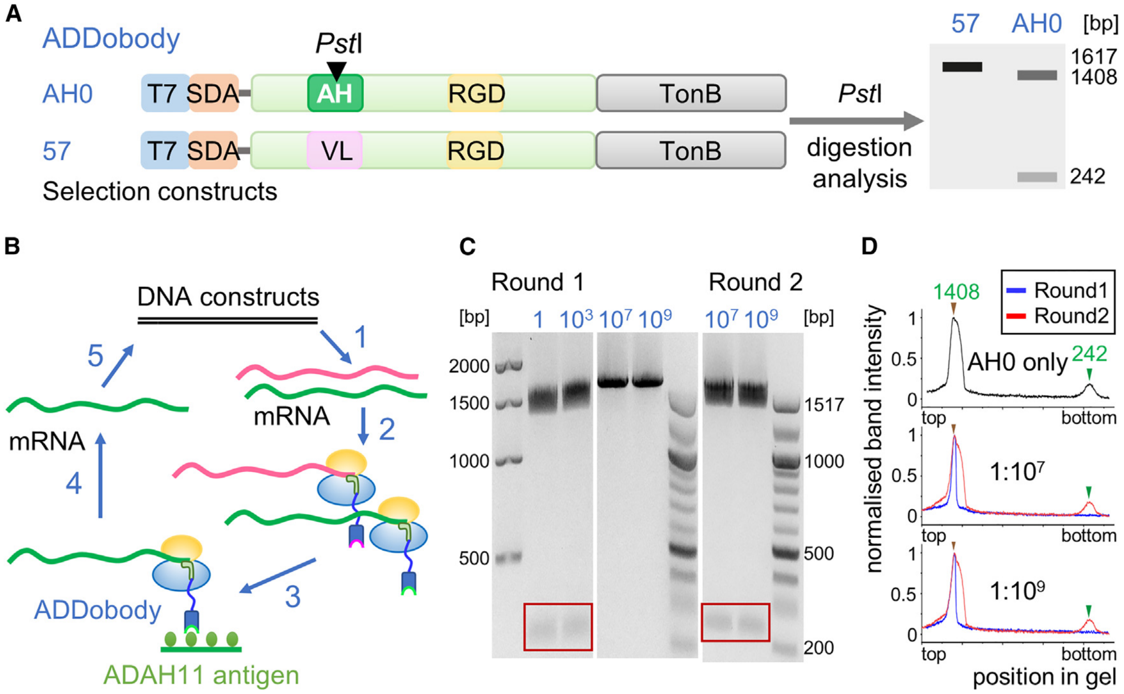

Figure 3. Proof-of-concept ribosome display selection using the ADDobody scaffold

(A) Schematic illustration of the ADDobody-encoding gene constructs used for ribosome display. Top: ADDobody-AH0 can bind ADAH11 and its encoding gene has a unique restriction site (*Pst*I). Bottom: The non-binding ADDobody-57. Right: *Pst*I restriction digest of these constructs yields two gene fragments (1408 and 242 bp) for ADDobody-AH0, but ADDobody-57 remains uncleaved.

(B) Schematic illustration of ADDobody ribosome display selection. Gene constructs encoding ADDobodies AH0 and 57 are transcribed *in vitro* (1). mRNA encoding ADDobody-AH0 is mixed with mRNA for ADDobody-57 in defined ratios (up to 1:10^9) for *in vitro* translation (2). Biotinylated ADAH11 is the bait for selections. Non-binding mRNA-ribosome-ADDobody complexes are washed away (3). The mRNA is eluted (4), reverse transcribed, and PCR amplified (5).

(C) Agarose gel of *Pst*I-digested PCR product after one and two rounds of ribosome display, monitoring enrichment of AH0 binders. Dilutions of AH0 into 57 are indicated in blue. The 242 bp fragment resulting from digestion of ADDobody-AH0 DNA is highlighted (red box).

(D) Quantification of *Pst*I digestion using DNA agarose gels showing that two rounds of ribosome display (red line) enrich ADDobody-AH0 from 1:10^7 and 1:10^9 dilutions. Upper panel: positive control (AH0 only, black line).

fold, with an RMSD of 0.49 Å and differences only found in the N-terminal region of the jelly-roll (Figure 4D). The N-terminal region of the PBP is essential for formation of stable dodecameric particles.[18] In the structure of the Chimera nanoparticle, the N-terminal regions comprising residues G47 to L59 of adjacent PBPs are observed to undergo strand swapping, stabilizing the interaction between pentons (Figure 4E). Strand swapping has been described before for human adenovirus Ad3 penton base dodecamers.[18] In contrast, in our previous cryo-EM structure of the Ad3 ADDomer, we observed a hairpin conformation of the N-terminal residues, and the interaction between the N-termini was stabilized by hydrogen bonds between S61 and the main chain of E59 (Figure 4E).[16] Notably, N-terminal residues 40–120 of Ad3 ADDomer and 36–116 of Chimera AH0 are identical in these constructs, but the penton-penton interactions are markedly different. In our structure, the penton-penton interface of Chimera AH0, the N-terminus of the PBP forms an anti-parallel β-sheet with a PBP from an adjacent penton, involving residues 50–54 and residues 106–110 (Figure 4F). In addition, polar contacts are formed between R48 and S113, N49 and D61, and R52 and D110. Y53 can interact with N94 and Q105, further stabilizing the penton-penton interface (Figure 4F).

In conclusion, we validated our Chimera nanoparticle design and demonstrate that "ADDomerization" of ADDobodies indeed results in picomolar super-binders against a given target.

DISCUSSION

The ADDomer nanoparticle has been used to develop vaccine candidates for diverse infectious diseases, displaying 60 and more B cell and T cell epitopes from SARS-CoV-2,[17,20] Chikungunya virus,[16] or type O foot-and-mouth disease.[30] Here, we engineered ADDobody to expand the scope of possible applications of the ADDomer nanoparticle, by converting it into a readily customizable, high-avidity super-binder against a target of choice. ADDobody is derived from the crown domain of the PBP, the protomer that self-assembles into ADDomer. Our ADDobody design retains the two hyper-variable VL and RGD loops that can be exploited for multi-epitope display for instance to yield vaccines, or, akin to antibody complementarity-determining regions, for recognizing and tightly binding a target molecule. We show that ADDobody can be expressed in *E. coli* at yields comparable to other scaffolds, is monomeric, and retains the thermostability observed for ADDomer (Figure 1C and 1E). We solved crystal structures of our ADDobody prototype at 2.9

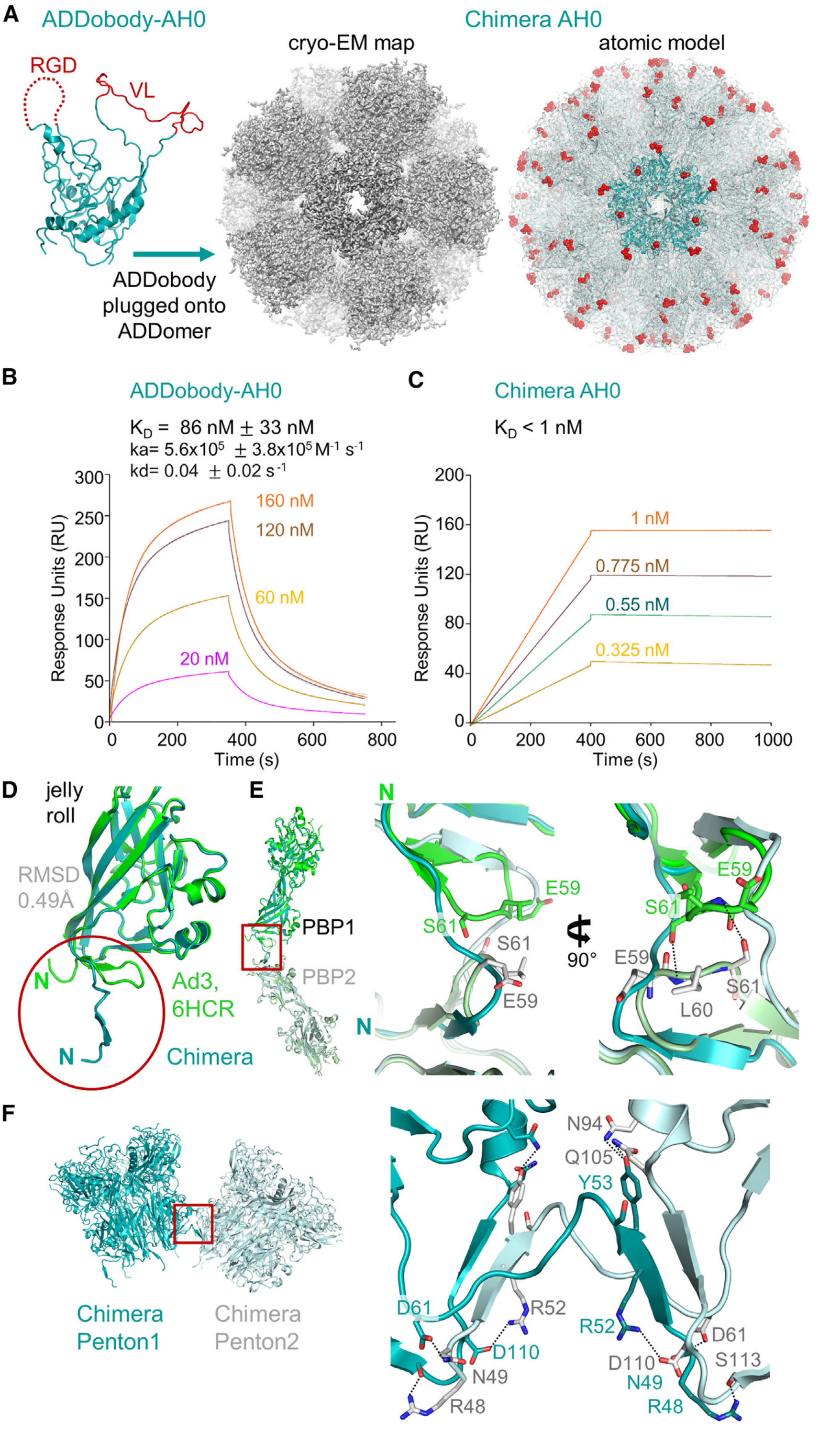

Figure 4. "ADDomerization" of ADDobody-AH0

(A) Right: ADDobody-AH0 (teal, loops in red) was conjoined with the jelly-roll fold domain of chimpanzee adenovirus Y25 penton base protein (PBP). Middle: Cryo-EM map of the resulting Chimera AH0. Left: Atomic model of Chimera AH0 (palegreen). One penton is shown in teal, loop residues are shown as red spheres.

(B) Surface plasmon resonance (SPR) of ADAH11 binding to ADDobody-AH0. Concentrations between 20 and 160 nM were flowed over 7,376 RU of ADDobody-AH0 immobilized on a CM5 sensor chip. A 1:1 binding model was used to calculate the K_D value (82 nM) and standard deviation.

(C) SPR of Chimera AH0. Concentrations between 0.325 and 1 nM were flowed over 1,137 RU of ADAH11 immobilized on a CM5 sensor chip. Fitting indicates a K_D in the picomolar range.

(D) Alignment of the jelly-roll folds of chimpanzee Y25 adenovirus PBP (teal) and ADDomer Ad3 adenovirus PBP (green, PDB: 6HCR[16]) with an RMSD of 0.371 Å. The N-termini are highlighted (red circle).

(E) Left: Alignment of PBPs from two adjacent pentons, highlighting the N-terminal region (red box). Middle and right: Close-up views of the N-terminal region, showing loop formation for the Ad3 PBPs (green and palegreen) and strand swapping for the Chimera PBPs (teal and palecyan). In Ad3, the S61 side chains form hydrogen bonds with the E59 peptide backbones.

(F) Close-up view of the penton-penton interface of Chimera AH0 (red box). An antiparallel β-sheet is formed between residues 50–54 of PBP1 and residues 106–110 of PBP2. Polar contacts formed between side chains are indicated (black dashed lines).

and 3.2 Å resolution. Our crystal structures confirm that ADDobody adopts the crown domain fold (Figures 2A and 2B) which is unique to adenovirus capsid protein according to the DALI protein structure comparison server.[31] Strikingly, ADDobodies form pentamers in the crystals indicating "molecular memory," implying that they actually may contribute to pentamerization of the PBPs which was thought to be mediated by the jelly-roll fold. In the crystals, ADDobody penton rings can stack on top of each other, forming decameric barrel shapes (Figure 2D and 2F) or form individual pentameric rings interacting with neighboring pentons via coordination of zinc ions supporting crystal packing (Figure 2G). Importantly, we show that ADDobodies can be used for ribosome display *in vitro* selections to enrich binders over non-binders (Figure 3). Given the high expression yields of ADDobodies in cells, we foresee their use in any other display or selection method on cells or within cells, e.g., phage display,[1] yeast surface display,[2] and yeast two-hybrid systems,[32] to select for binders. We estimate an average enrichment of ~10,000-fold per round of ribosome display for ADDobody-AH0. Similar experiments have been performed with single-chain variable domain (scFv) antibodies previously, to show that ribosome display is a *bona fide in vitro* selection method for binders.[33] Subsequently, it was shown that picomolar

binders can be selected and evolved *in vitro* starting from a synthetic scFv library with a size of $\sim 10^9$ members.[3] We confirmed that we can convert ADDobody-AH0 into a Chimera nanoparticle by fusing the crown domain with a jelly-roll domain from a chimpanzee adenovirus, as an example how ADDobody binders against a target of choice can be selected by ribosome display, and then converted into multimeric ultra-high avidity super-binders. The resulting Chimera nanoparticle assembled correctly and showed indeed high avidity as compared to monomeric ADDobody (Figures 4B and 4C), confirming the concept. The Chimera structure surprisingly showed stabilization of the dodecamer by N-terminal strand swapping between pentons, while our previous Ad3 ADDomer was stabilized by hairpin interaction[16] (Figures 4E and 4F). This finding cannot be explained by the amino acid residues directly involved in penton-penton interaction given that the sequences are identical in both. We speculate that long-range allosteric effects caused by the heterologous crown domain, derived from Ad3, subtle differences at the junctions between the crown domain and the jelly-roll fold, and the different VL and RGD loop sequences contribute to the distinct penton-penton interactions we observed.

We and others have shown that a range of peptides and small protein domains can be inserted into the VL and RGD loops of the PBP.[16,17,19,20,30] Moving forward, based on our results, we can now conceivably generate a synthetic ADDobody library with randomized VL and RGD loops that can be used for ribosome display *in vitro* selection and evolution experiments against any target of choice. The key advantage of ADDobody as a binder scaffold is that, by rejoining it with a jelly-fold roll domain, it can be readily converted back into a functional PBP which then self-assembles into the icosahedral nanoparticle comprising 12 pentons, for a total of 60 PBPs and thus 60 crown domains per nanoparticle. We demonstrated this here using an unrelated chimpanzee jelly-roll fold domain to generate a novel Chimera AH0 from our human Ad3-derived ADDobody-AH0 (Figure 4A). A 2.2 Å resolution cryo-EM structure confirmed successful assembly of the chimeric PBP into a dodecamer of pentons compellingly validating our design (Figures 4A, S8, S10, and S11). This "ADDomerization" generates 60 binding sites on the nanoparticle resulting in ultra-high avidity even when starting from moderate binding affinities—ADDobody-AH0 binds its ADAH11 target with a K_D of $\sim$82 nM, while Chimera AH0 binds ADAH11 with picomolar avidity with extremely slow dissociation kinetics, impressively illustrating the effect of 60 adjacent binding sites on a single particle. More generally, avidity is a useful means to enhance binding to a target, exploited by the immune system for instance by immunoglobulin G (IgG) antibodies possessing two binding sites, while IgA has four and IgM even ten binding sites for an antigen target. In protein engineering, multimerization has been used to generate multivalent nanobodies,[34,35] DARpins,[36] and antibodies,[37,38] often using binders that recognize different epitopes of the target. Typically, dimers, trimers, or tetramers were designed; however, antibody nanocages with up to 30 binding sites were also reported.[38]

How could our super-binders with 60 binding sites conceivably make a difference in the future? Ultra-high affinity/avidity is required for detection of toxins or chemicals, and for efficient clearance of toxins from organisms. We are particularly interested in snake toxins, and in innovating snakebite treatments, for use in particular in regions that are remote and do not afford a reliable cold chain. These regions are located mostly in low- and middle-income countries. Deployment of thermostable Chimera-based super-binders, that can be transported and stored independent of a cold chain, could provide a useful means over conventional IgG-based antivenoms in tropical areas where snakebite envenoming is a significant health, social, and economic burden. The World Health Organization declared snakebite envenoming as a neglected tropical disease and listed the generation of safe, effective, and affordable treatment as a key objective.[39,40] Based on our foundational work presented here, we anticipate that specific anti-toxin ADDobodies could be selected from a synthetic ADDobody library and converted into thermostable Chimera nanoparticles, to provide a basis of next-generation antivenom treatment.

STAR★METHODS

Detailed methods are provided in the online version of this paper and include the following:

- KEY RESOURCES TABLE
- RESOURCE AVAILABILITY
  - Lead contact
  - Materials availability
  - Data and code availability
- EXPERIMENTAL MODEL AND STUDY PARTICIPANT DETAILS
  - Microbe strains
  - Insect cell line
- METHOD DETAILS
  - Construct design and cloning
  - ADDobody expression and purification
  - Chimera nanoparticle expression and purification
  - ADDobody proof-of-concept ribosome display selection
  - Expression, purification, and biotinylation of ADAH11
  - Surface plasmon resonance assays
  - Thermal shift assays
  - Protein crystallization
  - Data collection and structure determination
  - Negative-stain EM
  - Cryo-EM and data collection
  - Image processing
  - Model building and refinement
- QUANTIFICATION AND STATISTICAL ANALYSIS

SUPPLEMENTAL INFORMATION

Supplemental information can be found online at https://doi.org/10.1016/j.str.2023.12.010.

ACKNOWLEDGMENTS

We thank all members, present and past, of the Berger and Schaffitzel laboratories for their contributions and helpful discussions. We are grateful for support from the Oracle Higher Education and Research program to enable cryo-EM data processing using Oracle's high-performance public cloud infrastructure (https://cloud.oracle.com/en_US/cloud-infrastructure), and we thank Simon Burbidge, Christopher Woods, Matt Williams, and Richard Pitts for computation infrastructure support. This work was carried out using the

computational and data storage facilities of the Advanced Computing Research Center, University of Bristol. The authors thank University of Bristol and the Max Planck Gesellschaft (MPG), Germany, for generous support through the Max Planck Bristol Center for Minimal Biology (MPBC). We acknowledge the staff of the BrisSynBio BioSuite including Peter Wilson and the beamline scientists at the Diamond Light Source (via BAG access) for their assistance with crystallographic studies. For the purpose of Open Access, the authors have applied a CC BY public copyright license to any Author Accepted Manuscript version arising from this submission.

We acknowledge support and assistance by the Wolfson Bioimaging Facility and the GW4 Facility for High-Resolution Electron Cryo-Microscopy funded by the Wellcome Trust (202904/Z/16/Z and 206181/Z/17/Z) and BBSRC (BB/R000484/1). I.B. acknowledges support from the EPSRC Future Vaccine Manufacturing and Research Hub (EP/R013764/1) and the ERC (AdvG 834631). C.S. and I.B. are Investigators of the Wellcome Trust (210701/Z/18/Z, 106115/Z/14/Z, and 221708/Z/20/Z). C.S. acknowledges funding by the Wellcome Trust – University of Bristol institutional Translation Partnership (iTP, fEC 282839). C.S. and I.B. are supported by an EC Horizon 2020 FET OPEN grant "ADDovenom" (contract no. 899670).

AUTHOR CONTRIBUTIONS

I.B. and C.S. conceived the study. D.B., H.S., I.B., and C.S. prepared figures and wrote the manuscript with help of all coauthors. D.B. purified ADDobody-Xtal and Chimera, performed crystallization trials, and solved the ADDobody-Xtal structure. D.B. processed cryo-EM data, determined the Chimera structure, and performed Biacore measurements with help from G.G.. H.S. performed ribosome display selections, purifications, and Thermofluor assays of ADDobodies. R.B.S. computationally designed the ADDobody prototype. F.G. and I.B. designed the Chimera construct. F.G. purified ADDobody and established the Chimera production protocol. J.C. purified Chimera. K.G. supported protein purification, crystallization, and data collection from zinc-free ADDobody crystals. J.C.B. and S.K.N.Y. supported image processing of Chimera. C.T. supported crystallization, data collection, structure solution, model building, and refinement of the X-ray and cryo-EM model. G.G supported Biacore measurements. U.B. performed cryo-EM data collection.

DECLARATION OF INTERESTS

C.S. and I.B. report shareholding in Halo Therapeutics Ltd. unrelated to this Correspondence. I.B. reports shareholding in Geneva Biotech SARL, unrelated to this Correspondence. I.B. and F.G. report shareholding in Imophoron Ltd., related to this Correspondence. Patents and patent applications have been filed related to ADDomer vaccines and therapeutics (WO2017167988A and EP22191583.8). ADDomer and ADDobody are registered trademarks of Imophoron Ltd.

Received: September 15, 2023
Revised: November 17, 2023
Accepted: December 14, 2023
Published: January 9, 2024

REFERENCES

1. Winter, G., Griffiths, A.D., Hawkins, R.E., and Hoogenboom, H.R. (1994). Making antibodies by phage display technology. Annu. Rev. Immunol. *12*, 433–455.
2. Gai, S.A., and Wittrup, K.D. (2007). Yeast surface display for protein engineering and characterization. Curr. Opin. Struct. Biol. *17*, 467–473.
3. Hanes, J., Schaffitzel, C., Knappik, A., and Plückthun, A. (2000). Picomolar affinity antibodies from a fully synthetic naive library selected and evolved by ribosome display. Nat. Biotechnol. *18*, 1287–1292.
4. Zimmermann, I., Egloff, P., Hutter, C.A.J., Kuhn, B.T., Bräuer, P., Newstead, S., Dawson, R.J.P., Geertsma, E.R., and Seeger, M.A. (2020). Generation of synthetic nanobodies against delicate proteins. Nat. Protoc. *15*, 1707–1741.
5. Dreier, B., and Plückthun, A. (2018). Rapid Selection of High-Affinity Antibody scFv Fragments Using Ribosome Display. Methods Mol. Biol. *1827*, 235–268.
6. Li, R., Kang, G., Hu, M., and Huang, H. (2019). Ribosome Display: A Potent Display Technology used for Selecting and Evolving Specific Binders with Desired Properties. Mol. Biotechnol. *61*, 60–71.
7. Zahnd, C., Spinelli, S., Luginbühl, B., Amstutz, P., Cambillau, C., and Plückthun, A. (2004). Directed *in vitro* evolution and crystallographic analysis of a peptide-binding single chain antibody fragment (scFv) with low picomolar affinity. J. Biol. Chem. *279*, 18870–18877.
8. Boder, E.T., Midelfort, K.S., and Wittrup, K.D. (2000). Directed evolution of antibody fragments with monovalent femtomolar antigen-binding affinity. Proc. Natl. Acad. Sci. USA *97*, 10701–10705.
9. Koenning, D., and Schaefer, J.V. (2021). Alternative Binding Scaffolds: Multipurpose Binders for Applications in Basic Research and Therapy. In Introduction to Antibody Engineering. Learning Materials in Biosciences, F. Rüker and G. Wozniak-Knopp, eds. (Springer).
10. Gebauer, M., and Skerra, A. (2020). Engineered Protein Scaffolds as Next-Generation Therapeutics. Annu. Rev. Pharmacol. Toxicol. *60*, 391–415.
11. Simeon, R., and Chen, Z. (2018). *In vitro*-engineered non-antibody protein therapeutics. Protein Cell *9*, 3–14.
12. Löfblom, J., Feldwisch, J., Tolmachev, V., Carlsson, J., Ståhl, S., and Frejd, F.Y. (2010). Affibody molecules: engineered proteins for therapeutic, diagnostic and biotechnological applications. FEBS Lett. *584*, 2670–2680.
13. Bloom, L., and Calabro, V. (2009). FN3: a new protein scaffold reaches the clinic. Drug Discov. Today *14*, 949–955.
14. Plückthun, A. (2015). Designed ankyrin repeat proteins (DARPins): binding proteins for research, diagnostics, and therapy. Annu. Rev. Pharmacol. Toxicol. *55*, 489–511.
15. Goux, M., Becker, G., Gorré, H., Dammicco, S., Desselle, A., Egrise, D., Leroi, N., Lallemand, F., Bahri, M.A., Doumont, G., et al. (2017). Nanofitin as a New Molecular-Imaging Agent for the Diagnosis of Epidermal Growth Factor Receptor Over-Expressing Tumors. Bioconjugate Chem. *28*, 2361–2371.
16. Vragniau, C., Bufton, J.C., Garzoni, F., Stermann, E., Rabi, F., Terrat, C., Guidetti, M., Josserand, V., Williams, M., Woods, C.J., et al. (2019). Synthetic self-assembling ADDomer platform for highly efficient vaccination by genetically encoded multiepitope display. Sci. Adv. *5*, eaaw2853.
17. Buzas, D., Bunzel, H.A., Staufer, O., Milodowski, E.J., Edmunds, G.L., Bufton, J.C., Vidana Mateo, B.V., Yadav, S.K.N., Gupta, K., Fletcher, C., et al. (2023). Antibodies Generated *in Vitro* and *in Vivo* Elucidate Design of a Thermostable ADDomer COVID-19 Nasal Nanoparticle Vaccine.
18. Szolajska, E., Burmeister, W.P., Zochowska, M., Nerlo, B., Andreev, I., Schoehn, G., Andrieu, J.P., Fender, P., Naskalska, A., Zubieta, C., et al. (2012). The structural basis for the integrity of adenovirus Ad3 dodecahedron. PLoS One *7*, e46075.
19. Villegas-Mendez, A., Garin, M.I., Pineda-Molina, E., Veratti, E., Bueren, J.A., Fender, P., and Lenormand, J.L. (2010). *In vivo* delivery of antigens by adenovirus dodecahedron induces cellular and humoral immune responses to elicit antitumor immunity. Mol. Ther. *18*, 1046–1053.
20. Chevillard, C., Amen, A., Besson, S., Hannani, D., Bally, I., Dettling, V., Gout, E., Moreau, C.J., Buisson, M., Gallet, S., et al. (2022). Elicitation of potent SARS-CoV-2 neutralizing antibody responses through immunization with a versatile adenovirus-inspired multimerization platform. Mol. Ther. *30*, 1913–1925.
21. Guo, M., Li, J., Teng, Z., Ren, M., Dong, H., Zhang, Y., Ru, J., Du, P., Sun, S., and Guo, H. (2021). Four Simple Biomimetic Mineralization Methods to Improve the Thermostability and Immunogenicity of Virus-like Particles as a Vaccine against Foot-and-Mouth Disease. Vaccines *9*.
22. Zochowska, M., Paca, A., Schoehn, G., Andrieu, J.P., Chroboczek, J., Dublet, B., and Szolajska, E. (2009). Adenovirus dodecahedron, as a drug delivery vector. PLoS One *4*, e5569.

23. Ashok, A., Brison, M., and LeTallec, Y. (2017). Improving cold chain systems: Challenges and solutions. Vaccine *35*, 2217–2223.

24. Besson, S., Vragniau, C., Vassal-Stermann, E., Dagher, M.C., and Fender, P. (2020). The Adenovirus Dodecahedron: Beyond the Platonic Story. Viruses *12*.

25. Shetty, R.P., Endy, D., and Knight, T.F., Jr. (2008). Engineering BioBrick vectors from BioBrick parts. J. Biol. Eng. *2*, 5.

26. Hoffmann, M., Kleine-Weber, H., Schroeder, S., Krüger, N., Herrler, T., Erichsen, S., Schiergens, T.S., Herrler, G., Wu, N.H., Nitsche, A., et al. (2020). SARS-CoV-2 Cell Entry Depends on ACE2 and TMPRSS2 and Is Blocked by a Clinically Proven Protease Inhibitor. Cell *181*, 271–280.e8.

27. Dupeux, F., Röwer, M., Seroul, G., Blot, D., and Márquez, J.A. (2011). A thermal stability assay can help to estimate the crystallization likelihood of biological samples. Acta Crystallogr. D Biol. Crystallogr. *67*, 915–919.

28. Schaffitzel, C., Zahnd, C., Amstutz, P., Luginbühl, B., and Plückthun, A. (2005). *In Vitro* Selection and Evolution of Protein-Ligand Interactions by Ribosome Display. In Protein-Protein Interactions A Molecular Cloning Manual, E. Golemis and P. Adams, eds. (Cold Spring Harbor Laboratory Press, Cold Spring Harbor), pp. 517–548.

29. Mattheakis, L.C., Bhatt, R.R., and Dower, W.J. (1994). An *in vitro* polysome display system for identifying ligands from very large peptide libraries. Proc. Natl. Acad. Sci. USA *91*, 9022–9026.

30. Luo, C., Yan, Q., Huang, J., Liu, J., Li, Y., Wu, K., Li, B., Zhao, M., Fan, S., Ding, H., and Chen, J. (2022). Using Self-Assembling ADDomer Platform to Display B and T Epitopes of Type O Foot-and-Mouth Disease Virus. Viruses *14*.

31. Holm, L. (2022). Dali server: structural unification of protein families. Nucleic Acids Res. *50*, W210–W215.

32. Brückner, A., Polge, C., Lentze, N., Auerbach, D., and Schlattner, U. (2009). Yeast two-hybrid, a powerful tool for systems biology. Int. J. Mol. Sci. *10*, 2763–2788.

33. Hanes, J., and Plückthun, A. (1997). *In vitro* selection and evolution of functional proteins by using ribosome display. Proc. Natl. Acad. Sci. USA *94*, 4937–4942.

34. Xiang, Y., Nambulli, S., Xiao, Z., Liu, H., Sang, Z., Duprex, W.P., Schneidman-Duhovny, D., Zhang, C., and Shi, Y. (2020). Versatile and multivalent nanobodies efficiently neutralize SARS-CoV-2. Science *370*, 1479–1484.

35. Wade, J., Rimbault, C., Ali, H., Ledsgaard, L., Rivera-de-Torre, E., Abou Hachem, M., Boddum, K., Mirza, N., Bohn, M.F., Sakya, S.A., et al. (2022). Generation of Multivalent Nanobody-Based Proteins with Improved Neutralization of Long alpha-Neurotoxins from Elapid Snakes. Bioconjugate Chem. *33*, 1494–1504.

36. Hanenberg, M., McAfoose, J., Kulic, L., Welt, T., Wirth, F., Parizek, P., Strobel, L., Cattepoel, S., Späni, C., Derungs, R., et al. (2014). Amyloid-beta peptide-specific DARPins as a novel class of potential therapeutics for Alzheimer disease. J. Biol. Chem. *289*, 27080–27089.

37. Leach, A., Miller, A., Bentley, E., Mattiuzzo, G., Thomas, J., McAndrew, C., Van Montfort, R., and Rabbitts, T. (2021). Implementing a method for engineering multivalency to substantially enhance binding of clinical trial anti-SARS-CoV-2 antibodies to wildtype spike and variants of concern proteins. Sci. Rep. *11*, 10475.

38. Divine, R., Dang, H.V., Ueda, G., Fallas, J.A., Vulovic, I., Sheffler, W., Saini, S., Zhao, Y.T., Raj, I.X., Morawski, P.A., et al. (2021). Designed proteins assemble antibodies into modular nanocages. Science *372*, eabd9994.

39. Harrison, R.A., and Gutiérrez, J.M. (2016). Priority Actions and Progress to Substantially and Sustainably Reduce the Mortality, Morbidity and Socioeconomic Burden of Tropical Snakebite. Toxins *8*, 351.

40. World Health Organisation (2021). Snakebite Envenoming. https://www.who.int/news-room/fact-sheets/detail/snakebite-envenoming.

41. Fitzgerald, D.J., Berger, P., Schaffitzel, C., Yamada, K., Richmond, T.J., and Berger, I. (2006). Protein complex expression by using multigene baculoviral vectors. Nat. Methods *3*, 1021–1032.

42. Conrath, K.E., Lauwereys, M., Galleni, M., Matagne, A., Frère, J.M., Kinne, J., Wyns, L., and Muyldermans, S. (2001). Beta-lactamase inhibitors derived from single-domain antibody fragments elicited in the *camelidae*. Antimicrob. Agents Chemother. *45*, 2807–2812.

43. Kabsch, W. (2010). XDS. Acta Crystallogr. D Biol. Crystallogr. *66*, 125–132.

44. McCoy, A.J., Grosse-Kunstleve, R.W., Adams, P.D., Winn, M.D., Storoni, L.C., and Read, R.J. (2007). Phaser crystallographic software. J. Appl. Crystallogr. *40*, 658–674.

45. Liebschner, D., Afonine, P.V., Baker, M.L., Bunkóczi, G., Chen, V.B., Croll, T.I., Hintze, B., Hung, L.W., Jain, S., McCoy, A.J., et al. (2019). Macromolecular structure determination using X-rays, neutrons and electrons: recent developments in Phenix. Acta Crystallogr. D Struct. Biol. *75*, 861–877.

46. Emsley, P., and Cowtan, K. (2004). Coot: model-building tools for molecular graphics. Acta Crystallogr. D Biol. Crystallogr. *60*, 2126–2132.

47. Williams, C.J., Headd, J.J., Moriarty, N.W., Prisant, M.G., Videau, L.L., Deis, L.N., Verma, V., Keedy, D.A., Hintze, B.J., Chen, V.B., et al. (2018). MolProbity: More and better reference data for improved all-atom structure validation. Protein Sci. *27*, 293–315.

48. Fernandez-Leiro, R., and Scheres, S.H.W. (2017). A pipeline approach to single-particle processing in RELION. Acta Crystallogr. D Struct. Biol. *73*, 496–502.

49. Zheng, S.Q., Palovcak, E., Armache, J.P., Verba, K.A., Cheng, Y., and Agard, D.A. (2017). MotionCor2: anisotropic correction of beam-induced motion for improved cryo-electron microscopy. Nat. Methods *14*, 331–332.

50. Rohou, A., and Grigorieff, N. (2015). CTFFIND4: Fast and accurate defocus estimation from electron micrographs. J. Struct. Biol. *192*, 216–221.

51. Yang, J., and Zhang, Y. (2015). I-TASSER server: new development for protein structure and function predictions. Nucleic Acids Res. *43*, W174–W181.

52. Pettersen, E.F., Goddard, T.D., Huang, C.C., Couch, G.S., Greenblatt, D.M., Meng, E.C., and Ferrin, T.E. (2004). UCSF Chimera–a visualization system for exploratory research and analysis. J. Comput. Chem. *25*, 1605–1612.

53. Barad, B.A., Echols, N., Wang, R.Y.R., Cheng, Y., DiMaio, F., Adams, P.D., and Fraser, J.S. (2015). EMRinger: side chain-directed model and map validation for 3D cryo-electron microscopy. Nat. Methods *12*, 943–946.

54. Sari-Ak, D., Bufton, J., Gupta, K., Garzoni, F., Fitzgerald, D., Schaffitzel, C., and Berger, I. (2021). VLP-factory and ADDomer((c)): Self-assembling Virus-Like Particle (VLP) Technologies for Multiple Protein and Peptide Epitope Display. Curr. Protoc. *1*, e55.

55. Estrozi, L.F., Boehringer, D., Shan, S.O., Ban, N., and Schaffitzel, C. (2011). Cryo-EM structure of the *E. coli* translating ribosome in complex with SRP and its receptor. Nat. Struct. Mol. Biol. *18*, 88–90.

56. Rosenthal, P.B., and Henderson, R. (2003). Optimal determination of particle orientation, absolute hand, and contrast loss in single-particle electron cryomicroscopy. J. Mol. Biol. *333*, 721–745.

STAR★METHODS

KEY RESOURCES TABLE

REAGENT or RESOURCE	SOURCE	IDENTIFIER
**Bacterial and virus strains**		
Escherichia coli BL21(DE3)	Thermo Fisher	#C600003
MultiBac baculovirus	Fitzgerald et al.[41]	N/A
Hi5 insect cells	Invitrogen	#B85502
**Chemicals, peptides, and recombinant proteins**		
Tryptone	Sigma	#1079-40-2
Yeast extract	Sigma	#8013-01-2
Isopropyl β-*d*-1-thiogalactopyranoside (IPTG)	Sigma	#367-93-1
COmplete EDTA-free protease inhibitor	Roche	#11836153001
Benzonase	Millipore	#E1014
KH_2PO_4	Sigma	#7778-77-0
TRIS hydrochloride	Sigma	#1185-53-1
HEPES	Sigma	#7365-45-9
Sodium citrate	Sigma	#6132-04-3
NaCl	Sigma	#7647-14-5
KCl	Sigma	#7447-40-7
Na-Acetate	Sigma	#127-09-3
Magnesium Acetate	Sigma	#16674-78-5
Zinc-Acetate	Sigma	#5970-45-6
Imidazole	Sigma	#288-32-4
Ethylene-dinitrilo-tetraacetic acid (EDTA)	Sigma	#60-00-4
Tween 20	Roche	#11332465001
NP-40 Detergent	Sigma	#9016-45-9
Glycine	Sigma	#56-40-6
PEG6000	Sigma	#81260
PEG3350	Sigma	#88276
2-Methyl-2,4-pentanediol (MPD)	Sigma	#107-41-5
Ethanolamine	Sigma	#141-43-5
Tobacco Etch Virus (TEV) protease	Home-purified clone	N/A
BirA Biotin Ligase	Home-purified clone	N/A
Streptavidin	Sigma	#9013-20-1
TranscriptAid RNA Kit	Thermo Fisher	#K0441
RNAeasy Kit	Qiagen	#74004
SuperScript IV cDNA Kit	Thermo Fisher	#11750150
Q5 DNA polymerase	New England Biolabs	#M0491
QIAquick Gel Extraction Kit	Qiagen	#28704
*Pst*I HF Restriction Enzyme	New England Biolabs	#R3140
Streptavidin Magnetic Beads	Thermo Fisher/Pierce	#88816
SuperBlock	Thermo Fisher	#37515
EDC/NHS Amine-Coupling Kit	Cytiva	#BR100050
SYPRO Orange	Thermo Fisher	#S6650
**Deposited data**		
ADDobody Xtal	This study	PDB ID: 8COI
ADDobody Xtal (with zinc)	This study	PDB ID: 8QB3
Chimera ADDomer AH0 map	This study	EMDB ID: EMD-18323

(*Continued on next page*)

Continued

REAGENT or RESOURCE	SOURCE	IDENTIFIER
Chimera ADDomer AH0 atomic model	This study	PDB ID: 8QBX
Oligonucleotides		
T7B_F_v3 [5′ ATACGAAATTAATACGACTCAC TATAGGGAGACCACAACGGTTTCCCTCTA GAAATAATTTTG 3’]	This study	N/A
A1.MS-SDA-ADDobody-F [5′ AGACCACAACGGT TTCCCTCTAGAAATAATTTTGTTTAACTTTAAG AAGGAGATATATATGGGATCCGGAATTCAACC 3’]	This study	N/A
tonBtot_R [5′CCGCACACCAGTAAGGTGTGC GGTCAGGATATTCACCACAATCCC 3’]	This study	N/A
A14-tonB-ADDobody-R [5′ CCGCACACCAGTA AGGTGTGCGGTCAGGATATTCAC 3’]	This study	N/A
Recombinant DNA		
pPROEX-HTB	Invitrogen	#10711018
pPROEX-HTB-ADDobody-Xtal	This study	N/A
pPROEX-HTB-ADDobody-AH0	This study	N/A
pPROEX-HTB-ADDobody-VL	This study	N/A
pPROEX-HTB-ADDobody-RGD	This study	N/A
pPROEX-HTB-ADDobody-VLRGD	This study	N/A
pPROEX-HTB-ADDobody-57	This study	N/A
pHEN6	Conrath et al.[42]	N/A
pHEN6-ADAH11_avi	This study	N/A
pHEN6-ADAH11	This study	N/A
pACEBac1	Geneva Biotech SARL, Switzerland[41]	N/A
pACEBac1-Chimera ADDomer	This study	N/A
Software and algorithms		
Biacore T200 Evaluation Software	Cytiva	https://www.cytivalifesciences.com/
XDS	Kabsch[43]	https://xds.mr.mpg.de/html_doc/XDS.html
PHASER	McCoy et al.[44]	https://www/ccp4.ac.uk
PHENIX version 1.17.1–3660	Liebschner et al.[45]	https://www/ccp4.ac.uk
Coot	Emsley & Cowtan[46]	https://www/ccp4.ac.uk
MolProbity	Williams et al.[47]	https://www/ccp4.ac.uk
PYMOL	Schreodinger, LLC	http://www.pymol.org
RELION 3.1	Fernandez-Leiro & Scheres[48]	https://www.ccpem.ac.uk/
MotionCorr2	Zheng et al.[49]	https://emcore.ucsf.edu/ucsf-software
Ctffind4.1	Rohou & Grigorieff[50]	https://grigoriefflab.umassmed.edu/ctffind4
I-TASSER	Yang & Zhang[51]	https://zhanggroup.org/I-TASSER/download/
UCSF Chimera software	Pettersen et al.[52]	https://www.cgl.ucsf.edu/chimera/download.html
EMRinger	Barad et al.[53]	https://github.com/fraser-lab/EMRinger
Other		
Mono Q 5/50 GL column	Cytiva	#17516601
Superdex 200 10/300 GL column	Cytiva	#17517501
Superdex 200 26/100 column	Cytiva	#90100273
Bio-Scale Mini Macro Prep High Q	Bio-Rad	#7324120
HiTrap Capto Q column	Cytiva	#11001302
HisPur Cobalt resin	Thermo Fisher	#89964

(*Continued on next page*)

Continued

REAGENT or RESOURCE	SOURCE	IDENTIFIER
Amicon centrifugal ultrafiltration units MWCO 10 kDa.	Millipore	#UFC5010
Amicon centrifugal ultrafiltration units MWCO 50 kDa.	Millipore	#UFC8100
CM5-Series S sensor chip	Cytiva	#BR100530
Cu 300 mesh carbon grids	Sigma Aldrich	#TEM-HC300CU100
Quantifoil R 1.2/1.3 grids	EMS	#Q225CR-06

RESOURCE AVAILABILITY

Lead contact

Further information and requests for resources and reagents should be directed to the lead contact, Christiane Schaffitzel (cb14941@bristol.ac.uk).

Materials availability

Plasmids generated in this study are available from the lead contact upon reasonable request. This study did not generate other new or unique reagents.

Data and code availability

- All X-ray diffraction data, the EM map and model have been deposited in the PDB and EMDB. They are publicly available as of the date of publication. Accession numbers are listed in the key resources table.
- This paper does not report original code.
- Any additional information required to reanalyze the data reported in this paper is available from the lead contact upon request.

EXPERIMENTAL MODEL AND STUDY PARTICIPANT DETAILS

Microbe strains

ADDobody Protein expression was done in BL21(DE3) chemically competent E. coli cells from ThermoFisher Scientific (#C600003).

Insect cell line

CHIMERA was expressed in *Trichoplusia ni* Hi5 insect cells.from Invitrogen (#B85502).

METHOD DETAILS

Construct design and cloning

Based on structural data of the Ad3 adenovirus PBP (PDB: 4AQQ), a DNA sequence encoding the crown domain encompassing residues 132–461 was codon-optimized for *E.coli* BL21(DE3) expression, and a 6 residue long, flexible linker encoding NGDSGN sequence was introduced to reconnect the crown domain replacing two β-strands from the jelly-roll fold domain (Figure 1B). ADDobody constructs were gene-synthesized (Genscript) and ligated into the pPROEX-HTB vector (Invitrogen) using *Eco*RI and *Not*I restriction sites. ADDobody-Xtal construct was optimized for crystallization by minimizing the VL and RGD flexible loop regions from 12 to 5 residues and 45 to 4 residues, respectively. The ADDobody-AH0 construct contained the SARS-CoV-2 Spike protein RBM-derived AH0 sequence YQAGSTPCNGVEGFNCYFPLQSYGFQPTNGVGY, inserted into the VL loop region.[17] To facilitate insertion of sequences into the loop regions, we adopted a BioBrick design with restriction sites marking the boundaries of the VL loop (*Eco*RI and *Rsr*II) and the RGD loop (*Bss*HII and *Xba*I) (Figure 1).[16] The amino acid sequences of all ADDobodies used in this study are provided in Figure S1.

The Chimera construct was designed to comprise the crown domain representing ADDobody and the jelly-roll domain of the chimpanzee derived adenovirus Y25 PBP (UniProt-ID: G9G849) comprising an A57S mutation. Furthermore, we implemented mutations in the Chimera nanoparticle to conserve the metal-ion cluster we identified previously in the human Ad3 derived ADDomer.[16] The resulting gene encoding for the Chimera PBP was synthesized (Genscript), and inserted into plasmid pACEBac1 (Geneva Biotech SARL, Switzerland) using *Bam*HI and *Hind*III restriction sites, giving rise to pACEBac-Chimera.

ADDobody expression and purification

Plasmids encoding the individual ADDobody constructs were transformed into *E. coli* BL21(DE3) (Thermo Fisher) and grown in 2xYT media. At an OD_{600nm} of 0.9, protein expression was induced with 1 mM isopropyl β-*d*-1-thiogalactopyranoside (IPTG) for 3 h at 30°C with agitation. The cells were harvested by centrifugation at 4000 × g for 15 min at 4°C. The supernatant was removed, and the pellets were flash-frozen in liquid nitrogen, followed by storage at −80°C.

For purification, cell pellets from 1 L culture were resuspended in 20 mL 1× phosphate-buffered saline (PBS), lysed via sonication for 10 min with pulse settings (10 s on, 15 s off) at 70% amplitude while lysate was chilled on ice, and the lysate clarified at 40,000 × g for 15 min at 4°C. The supernatant was applied to 2 mL HisPur Cobalt resin (Thermo Fisher) and incubated for 16 h at 4°C while gently mixed by rotation. After washing the resin with high-salt wash buffer (50 mM Tris, 1 M KCl, 5 mM imidazole pH 7.4) and 1× PBS, proteins were eluted using 1× PBS buffer with 200 mM imidazole. Eluted protein-containing fractions were dialyzed into 1× PBS with 2 mM beta-mercapto-ethanol while incubated with 0.5 mg Tobacco Etch Virus (TEV) protease for the removal of the 6xHis-tag. The proteins were further purified by reverse immobilized metal ion affinity chromatography (IMAC) using TALON resin (Qiagen), to remove uncleaved protein, followed by ion exchange purification using a 5 mL Mono Q 5/50 GL column (Cytiva) equilibrated in Buffer A (1× PBS, pH 7.4) and proteins were eluted by using gradient buffer B (from 0.1 M to 1 M NaCl in 1× PBS, pH 7.4). Subsequently, SEC with a Superdex 200 10/300 GL column (Cytiva) equilibrated with 1× PBS, pH 7.4 was used to further purify proteins. Proteins were concentrated using Amicon centrifugal ultrafiltration units with a molecular weight cut-off of 10 kDa. Protein concentrations were determined by measuring UV absorbance at 280 nm with calculated molecular weights and extinction coefficients based on ProtParam EXPASY tool (https://web.expasy.org/protparam/) using a NanoDrop One spectrophotometer (Thermo Fisher). Aliquots of purified protein in 1x PBS, pH 7.4 were flash-frozen in liquid nitrogen and stored at −80°C.

Chimera nanoparticle expression and purification

Chimera was expressed using the MultiBac baculovirus and Hi5 insect cells following established protocols[54] and purified as described previously.[17] Briefly, pellets were resuspended in Resuspension Buffer (50 mM Tris pH 7.5, 150 mM NaCl, 2 mM $MgCl_2$, 1 mL per 2.5×10^7 cells) supplemented by EDTA-free complete protease inhibitor (Roche). Lysate was prepared by three cycles of freeze-thawing, cleared by centrifugation (40,000g, 30 min), supplemented with Benzonase (Sigma-Aldrich) and incubated on ice for 2 h. Precipitate was removed by centrifugation (4000 × g, 15 min), the supernatant passed through a 0.45 μm filter, and subjected to SEC using an XK 26/100 column (GE Healthcare) equilibrated with 50 mM Tris pH 7.5, 150 mM NaCl running buffer. Fractions containing Chimera were pooled and further purified by ion-exchange chromatography (IEX) using a 5 mL Bio-Scale Mini Macro Prep High Q (Bio-Rad) equilibrated in Buffer A (50 mM Tris pH 7.5, 150 mM NaCl) and a linear salt gradient from 0.15 M to 1 M NaCl. Highly purified Chimera eluted at ~250 mM–400 mM NaCl. Fractions were pooled, concentrated using Amicon centrifugal ultrafiltration units with a molecular weight cut-off of 50 kDa, and the buffer was exchanged to 50 mM Tris pH 7.5, 150 mM NaCl. Aliquots were flash-frozen in liquid nitrogen and stored at −80°C.

ADDobody proof-of-concept ribosome display selection

Ribosome display selections were carried out as described previously.[28] Briefly, the gene fragments encoding ADDobody-AH0 and ADDobody-57 were converted into ribosome display format by adding a C-terminal spacer sequence derived from the periplasmic part of tonB from *E. coli*[3] and N-terminal ribosome binding site. 1 μg of ADDobody-AH0 and ADDobody-57 DNA in ribosome display format were transcribed *in vitro* using the TranscriptAid RNA kit (Thermo Fisher). The mRNA was purified using the RNAeasy kit (Qiagen). *E. coli* cell extract for *in vitro* translation was prepared as described.[55] ADDobody-AH0 RNA was diluted into ADDobody-57 RNA using different ratios (1,1:1, 1:10^3, 1:10^5, 1:10^7, and 1:10^9). A total of 10 μg RNA mixtures was used in *in vitro* translation reactions which were performed as described.[3] Next, translation products in solution were incubated with 100 nM biotinylated antigen (ADAH11-Bio). In negative control reactions, no antigen was added in translation products which contain only ADDobody-AH0 or ADDobody-57. After 60 min of incubation at 4°C, the antigens were captured for 15 min at 4°C with 50 μL Streptavidin Magnetic beads (Thermo Fisher) which were previously incubated with superblock (Thermo Fisher) for around 1 h. After 6 washing steps (50 mM Tris-acetate, 150 mM NaCl, 50 mM magnesium acetate, 0.1% Tween 20, pH 7.5 at 4°C), the mRNA was eluted with EDTA-buffer (50 mM Tris-acetate, 150 mM NaCl, 25 mM EDTA, pH 7.5 at 4°C) and purified using the RNAeasy kit (Qiagen).

Subsequently, the mRNA was immediately reverse transcribed using the SuperScript IV cDNA kit (Thermo) and primer tonBtot_R (Table S2). The resulting cDNA was PCR amplified using primers A1.MS-SDA-ADDobody-F and tonBtot_R (Table S2) using Q5 DNA polymerase (New England Biolabs) and reactions conditions according to the manufacturer's recommendations (initial denaturation at 98°C for 30 s, followed by 30 cycles of: 98°C for 10 s, 68°C for 20 s, 72°C for 30 s, and a final extension at 72°C for 2 min). Then, the PCR product was gel-extracted using QIAquick Gel Extraction Kit (Qiagen). This was followed by a second PCR amplification step with primers T7B_F_v3 and A14-tonB-ADDobody-R (same PCR program as described above) (Table S2). The final PCR product was gel extracted and used for the next round of *in vitro* transcription and selection.

A unique restriction site (*Pst*I) is present in the gene fragment encoding ADDobody-AH0 (in ribosome display selection format), but not in the ADDobody-57 construct. Therefore, *Pst*I was used to digest the PCR product after ribosome display selection rounds to test if the AH0 construct was enriched. *Pst*I digestion of AH0 generates 2 fragments (242 bp and 1390 bp) (Figure 3A). 1 μg of purified DNA (PCR product after 2nd round PCR after selections) was incubated with *Pst*I-HF (New England Biolabs) at 37°C for 1 h. Subsequently, the reaction was analyzed by agarose gel electrophoresis.

Expression, purification, and biotinylation of ADAH11

The gene encoding Avi-tagged ADAH11 (ADAH11-Avi) was cloned into the plasmid pHEN6,[42] which encodes a C-terminal 3xFLAG and 6xHis tag and an N-terminal pelB signal sequence for the expression of proteins in the bacterial periplasm. For expression, the plasmid pHEN6_ADAH11 was transformed into the *E. coli* BL21(DE3) strain. Cells were grown in 2xYT media at 37°C until OD_{600} around 0.8, and protein expression was induced by 1 mM IPTG at 30°C overnight. Cells were harvested by centrifugation

(3200xg, 15 min) and, to extract the bacterial periplasm, cell pellets were resuspended in 10 mL cold TES buffer (50 mM Tris pH 8.0, 20% Sucrose, 1 mM EDTA) supplemented with EDTA-free complete protease inhibitor (Roche, Switzerland) and incubated at 4°C for 45 min. Subsequently, 15 mL of ice-cold shock buffer (20 mM Tris pH 8.0, 5 mM $MgCl_2$) was added and incubated at 4°C for 45 min, followed by centrifuging at 13,000xg for 30 min at 4°C. The supernatant was kept as the periplasm. The supernatant containing the periplasm was applied to 2 mL HisPur Cobalt resin (Thermo Fisher) and incubated for 2 h at 4°C. After washing the resin with wash buffer 1 (50 mM HEPES, 200 mM KCl, 10 mM imidazole pH 8.0) and wash buffer 2 (50 mM HEPES, 200 mM KCl, 20 mM imidazole pH 8.0), the fractions of ADAH11-Avi were eluted in elution buffer (50 mM HEPES, 200 mM KCl, 500 mM imidazole pH 8.0). The purified protein was dialyzed into PBS, pH 7.4, at 4°C overnight (Figure S6).

In order to biotinylate ADAH11-Avi, 30 μM ADAH11-Avi protein was incubated with 2 μM maltose binding protein-tagged biotin ligase BirA in PBS buffer containing 5 mM $MgCl_2$, 5 mM ATP, and 150 μM biotin at 4°C overnight. Afterward, size exclusion chromatography was performed using a Superdex 75 pg column (GE Healthcare Life Sciences) to remove excess BirA protein, biotin and to further purify ADAH11-Avi. Anion exchange chromatography (HiTrap Capto Q column) was conducted to further purify biotinylated ADAH11 and to remove any RNases using PBS pH7.4 buffer and applying a linear salt (from 0.1 M to 1 M NaCl) gradient. Biotinylated ADAH11-Avi (ADAH11-Bio) eluted at ~400 mM NaCl.

To determine the biotinylation efficiency, a gel shift assay using streptavidin (Sigma) was performed. Biotinylated ADAH11 was pre-incubated with streptavidin and loaded onto an SDS-gel, resulting in a shift of the ADAH11-Bio protein to higher molecular weight. As a control, a sample without streptavidin was loaded onto the same SDS-gel (Figure S6).

Surface plasmon resonance assays

Surface plasmon resonance (SPR) experiments were performed on a Biacore T200 using a CM5-Series S sensor chip (Cytiva). Freshly prepared, filtered, and degassed HBS-P+ running buffer (10 mM HEPES pH 7.4, 150 mM NaCl and 0.05% v/v NP40) was used at 25°C. ADDobody-AH0 was immobilized by 1-Ethyl-3-[3-dimethylaminopropyl] carbodiimide hydrochloride (EDC)/N-hydroxy succinimide (NHS) chemistry, using an amine-coupling kit (Cytiva). A flow cell pair (Fc1 and Fc2) was activated using the amine coupling kit, and immobilization of the protein was carried out on a single flow cell leaving a second flow cell as a background control for signal subtraction. The chip surface was activated by injecting a 1:1 (v/v) mixture of 200 mM EDC and 50 mM NHS for 7 min 30 μg purified ADDobody-AH0 in 10 mM Na-acetate pH 4.5 at a concentration of 300 μg/mL was injected for 10 min, followed by injection of 100 mM ethanolamine pH 8.5 for 1 min leading to surface inactivation. After immobilization, ~7400 response units (RU) were reached.

ADAH11 binding to ADDobody-AH0 was measured using concentrations ranging from 20 nM to 160 nM in HBS-P+ running buffer. Samples were injected at 30 μL/min with an association phase of 400 s and a dissociation phase of 400 s. After every experiment, the surface was regenerated with a 60 s injection regeneration solution (10 mM glycine pH 2.0).

For Chimera AH0 binding experiments, 25 μg purified ADAH11 in 10 mM Na-acetate pH 4.5 at a concentration of 250 μg/mL was immobilized as outlined above for ADDobody-AH0. After immobilization, ~1100 response units (RU) were reached. Chimera AH0 binding to ADAH11 was tested using concentrations ranging from 0.325 nM to 1 nM in HBS-P+ running buffer. Samples were injected at 30 μL/min with an association phase of 400 s and a dissociation phase of 600 s. After every experiment, the surface was regenerated with a 60 s injection of regeneration solution (10 mM glycine pH 1.5). Sensorgrams were analyzed with the Biacore Evaluation software (version 1.0) yielding on- and off-rates obtained through fitting the association and dissociation phases of four different concentrations of each analyte, each measured 3 times (n = 3), using the one-to-one binding model.

Thermal shift assays

Thermal shift experiments on highly purified Chimera nanoparticle and ADDobody constructs were performed using a ThermoFluor assay.[27] ADDobody and Chimera samples were diluted to 1 mg/mL in 1 × PBS buffer and supplemented with 1 × SYPRO Orange probe in 25 μL reaction volumes (Thermo Fisher). Samples were subjected to thermal denaturation in a Real-Time PCR machine (Agilent Stratagene Mx3005P). Fluorescence intensity of the probe was recorded throughout denaturation using a temperature gradient from 25°C to 95°C, with a step size of 1°C per minute, using excitation and emission wavelengths of 492 nm and 516 nm, respectively. Relative fluorescence emission intensity (R) was plotted as a function of the temperature and normalized to range between 0 and 1. The melting temperature (Tm) was determined as the temperature corresponding to the midpoint between the baseline and the point with maximum fluorescence intensity.

Protein crystallization

Crystallization experiments were performed with ADDobody-Xtal using a standard sitting drop vapor diffusion method on MRC 2 Well Crystallization Polystyrene plate (Swissci) containing 60 μL of reservoir solution. Using a Mosquito crystallization robot (SPT Labtech) 150 nL and 175 nL of 5 mg/mL ADDobody-Xtal in 1x PBS buffer was dispensed along with 150 nL and 125 nL of reservoir solution in drops A and B respectively resulting in 300 nL total drop volume. Plates were incubated at 20°C. The best diffracting crystals appeared after 7 days in 20% (w/v) PEG6000, 0.1 M zinc-acetate, 0.1 HEPES pH 7.0 for zinc-containing ADDobody; and after 10 days in 20% (w/v) PEG3350, 0.15 M citrate pH 5.5 for zinc-free ADDobody. Crystals were transferred in a solution containing 20% 2-Methyl-2,4-pentanediol (MPD) as a cryoprotectant in the crystallization condition prior to flash freezing in liquid N_2.

Data collection and structure determination

Diffraction data were collected on the I03 (zinc-free ADDobody) and I04 beamlines (zinc-containing ADDobody) at the Diamond Light Source under nitrogen cryo-stream (~100 K) (Harwell Science and Innovation Campus) and images processed using XDS (version 03/2019) and scaled with AIMLESS[43] (Table 1). Structures were solved by molecular replacement in PHASER using the Ad3 PBP crown domain structure (PDB ID: 4AQQ) as an input model.[16] Manual model building was performed in Coot (version 0.8.9.2) and refinement carried out in PHENIX.REFINE (PHENIX version 1.17.1–3660).[45,46] Models were validated and statistics obtained using MolProbity.[47] Figures were prepared using PyMol (version 4.6.0).

Negative-stain EM

Negative-stain EM quality control was performed with purified Chimera (0.1 mg/mL) in 50 mM TRIS (pH 7.5), 150 mM NaCl. 4 μL sample was applied onto glow discharged Cu 300 mesh carbon grids before staining with 2% (w/v) uranyl acetate. Images were acquired with a 120 kV FEI Tecnai 12 electron microscope equipped with a Ceta camera (Thermo Fisher).

Cryo-EM and data collection

4 μL of purified Chimera (0.5 mg/mL) was applied onto carbon-coated Quantifoil R 1.2/1.3 grids (Sigma-Aldrich) which previously were glow discharged for 120 s at 5 mA. Excess protein was blotted away for 2 s at 4°C in 100% relative humidity before being plunge frozen into liquid ethane using a Vitrobot Mark IV (Thermo Fisher). Cryo-EM data were collected with a 200 kV FEI Talos Arctica microscope equipped with a Gatan K2 direct electron detector and an energy filter using automated acquisition software (EPU). In total, 6,179 dose-fractioned movies were recorded in super-resolution mode each containing 40 frames with an accumulated total dose of 42.4 e/$Å^2$ recorded in super-resolution mode at a nominal magnification of 130,000× corresponding to a physical pixel size of 1.05 Å and a virtual pixel size of 0.525 Å. Images were recorded with a defocus range of −0.8 to −2.0 μm (Table S1).

Image processing

Image processing was performed with the RELION 3.1 software package.[48] The micrographs were motion-corrected using MotionCorr2,[49] and contrast transfer function (CTF) information was determined using ctffind4.1.[50] Micrographs with significant astigmatism (>500) and defocus (>2.1 μm) were removed. This resulted in the selection of 5,474 micrographs with CTF rings extending below 4.0 Å that were used for further processing. A total of 710,760 particles were boxed using RELION autopicking software. Several rounds of reference-free 2D classification were performed (Figure S10) yielding 566,795 particles which were subjected to 3D classification using a previous ADDomer structure[16] filtered to 60 Å as initial 3D model. Class 1 (of two) comprised 377,978 good particles which were subjected to 3D refinement yielding a reconstruction of ~2.6 Å resolution. Particles were further subjected to Bayesian polishing and CTF refinement and final 3D refinement with imposed icosahedral symmetry. Subsequently, postprocessing was performed for masking and automatic B-factor sharpening with a B-factor value of −88. The resolution of the final map was determined to be 2.2 Å based on the Fourier shell correlation (FSC) 0.143 cutoff criterion[56] (Figure S11A). Local resolution was calculated using the local resolution estimation program in RELION (Figure S11B). Image processing and 3D classification of Chimera nanoparticle were performed using public cloud resources provided by the Oracle Cloud Infrastructure.

Model building and refinement

Model building was performed using I-TASSER[51] with the Ad3-derived ADDomer structure (PDB ID: 6HCR)[16] as template. The generated model was manually docked into the density map in UCSF Chimera software.[52] Refinement was performed using Phenix Real-Space refinement software version 1.17[45] and COOT[46] for model-building, before evaluating the model using MolProbity[47] and EM-Ringer.[53] Refinement statistics are summarized in Tables 1 and S1.

QUANTIFICATION AND STATISTICAL ANALYSIS

Statistical calculations for X-ray structure analysis were carried out with PHENIX.REFINE (PHENIX version 1.17.1–3660) and Molprobity and are reported in Table 1. Statistical calculations for cryo-EM reconstruction and structural analysis were done in RELION 3.1, MolProbity and EMRinger and are reported in Table S1. Statistical analyses for the surface plasmon resonance (SPR) experiments were carried out with the Biacore Evaluation software (version 1.0) and in Microsoft Excel.

Structure, Volume *32*

Supplemental Information

Engineering the ADDobody protein scaffold for generation of high-avidity ADDomer super-binders

Dora Buzas, Huan Sun, Christine Toelzer, Sathish K.N. Yadav, Ufuk Borucu, Gunjan Gautam, Kapil Gupta, Joshua C. Bufton, Julien Capin, Richard B. Sessions, Frederic Garzoni, Imre Berger, and Christiane Schaffitzel

Supplementary Information

Engineering the ADDobody protein scaffold for generation of high-avidity ADDomer super-binders

Dora Buzas[1,2,6], Huan Sun[1,2,3,6], Christine Toelzer[1], Sathish K. N. Yadav[1], Ufuk Borucu[1], Gunjan Gautam[1], Kapil Gupta[1,4], Joshua C. Bufton[1], Julien Capin[1], Richard B. Sessions[1], Frederic Garzoni[4], Imre Berger[1,2,5]* & Christiane Schaffitzel [1]*

[1] School of Biochemistry, University of Bristol, University Walk, Bristol BS8 1TD, UK

[2] Max Planck Bristol Centre for Minimal Biology, Cantock's Close, Bristol BS8 1TS, UK

[3] Present address: The Institute of Medicinal Plant Development (IMPLAD), No. 151 Malianwa North Road, Haidian District, Beijing 100193, P. R. China

[4] Imophoron Ltd, Science Creates Old Market, Midland Rd, Bristol BS2 0JZ, UK

[5] School of Chemistry, University of Bristol, Cantock's Close, Bristol BS8 1TS, UK

[6] These authors contributed equally.

* Correspondence: Christiane Schaffitzel, 0044 (0)117 394 1869; cb14941@bristol.ac.uk

Imre Berger, 0044 (0)117 394 1857; imre.berger@bristol.ac.uk

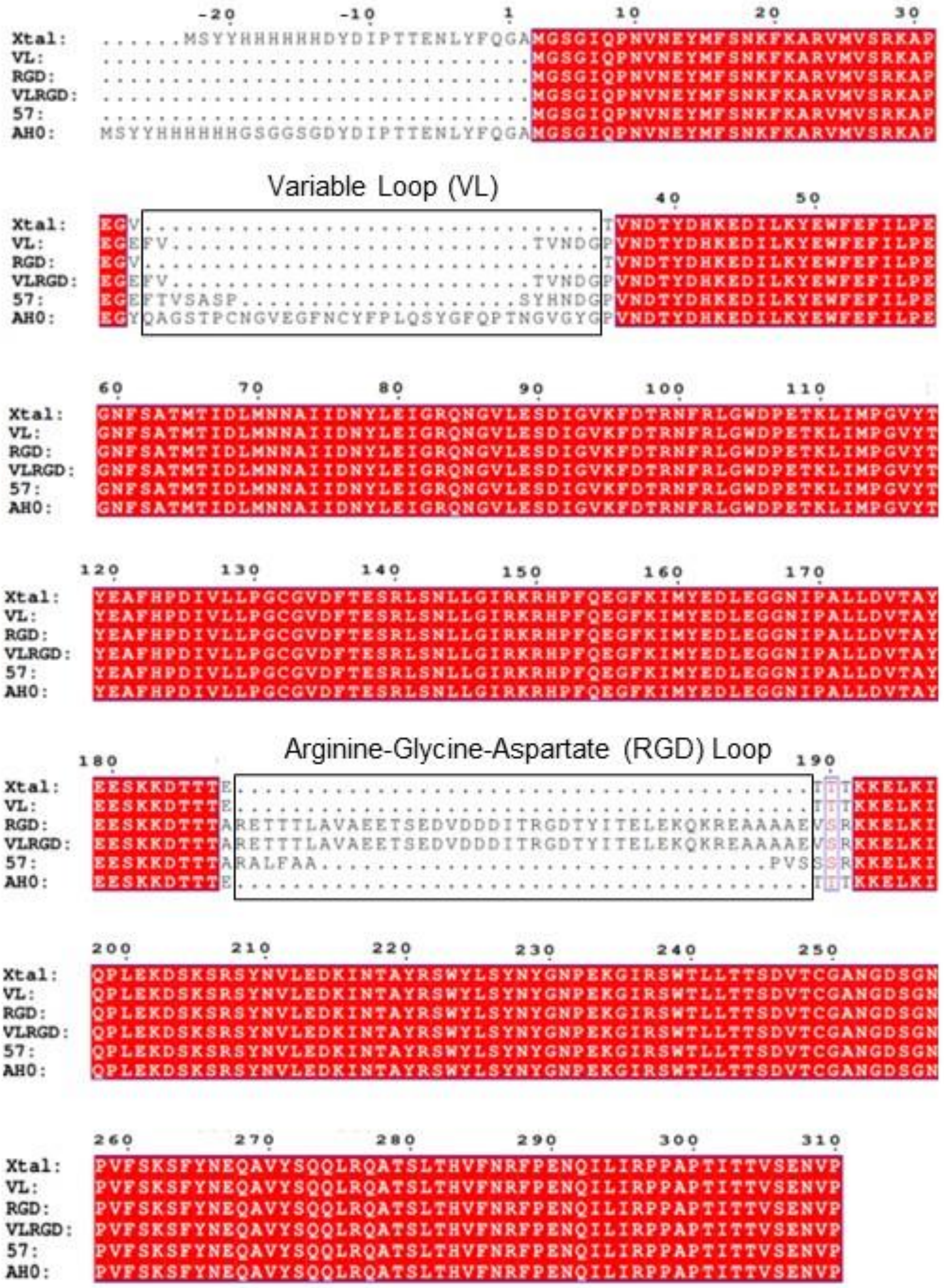

Figure S1: Amino-acid sequences of ADDobodies used in this study, Related to Figure 1, 2 and 3.

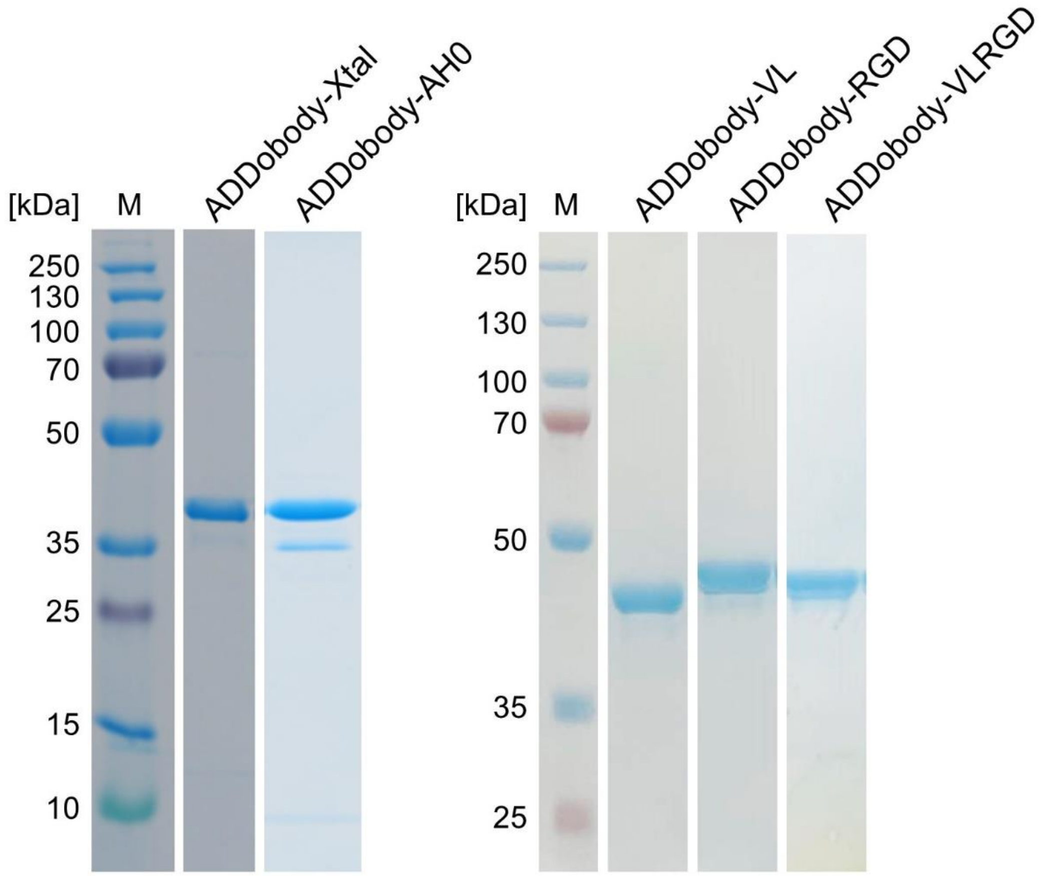

Figure S2. Coomassie-stained SDS gel showing purified ADDobodies, Related to Figure 1. Coomassie-stained SDS–PAGE showing purified ADDobody constructs. M: Molecular weight marker. The sequence of the ADDobodies is provided in Fig. S1.

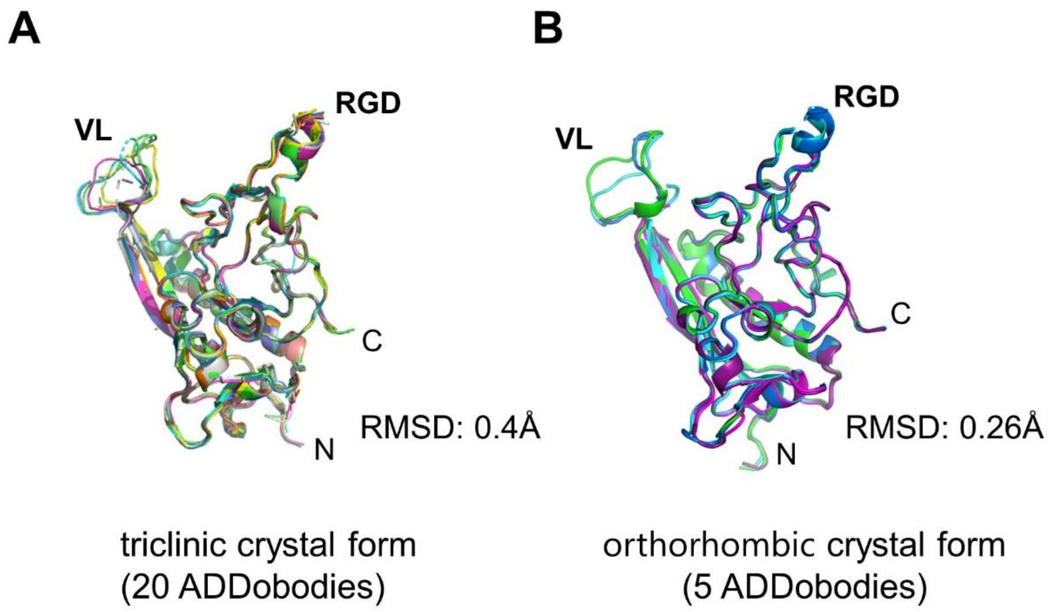

Figure S3. Superimposition of ADDobodies, Related to Figure 2. **(A)** from the triclinic (P1) crystal form comprising 20 ADDobody molecules in the asymmetric unit, adopting four pentons forming two decamer barrels, and **(B)** from the Zn^{2+}-containing orthorhombic ($P2_12_12_1$) crystal form (5 ADDobodies per asymmetric unit adopting a penton). The root-mean-square deviation (RMSD) of the overlay is indicated.

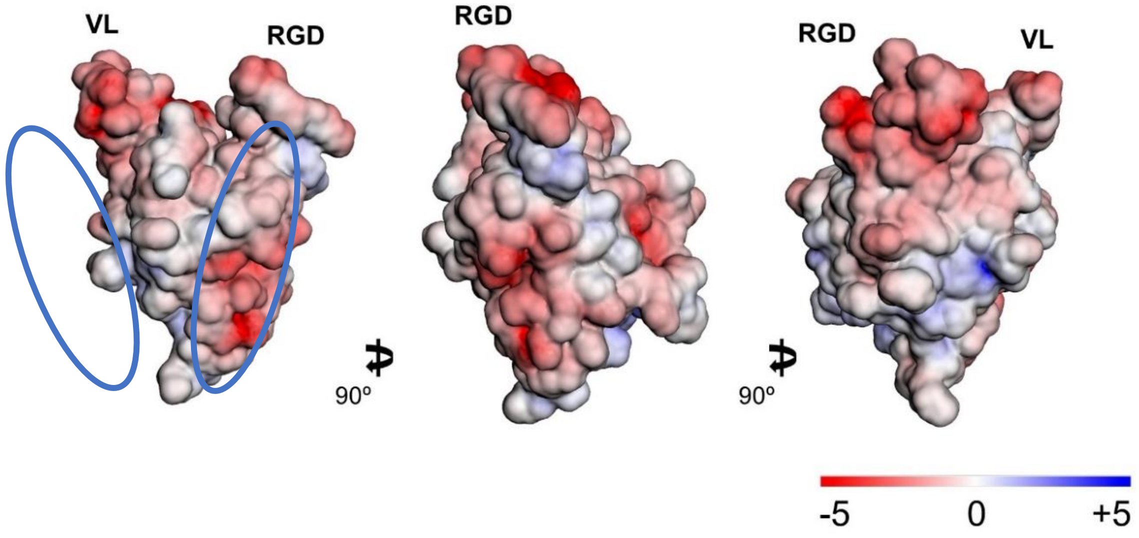

Figure S4. Electrostatic (Coulomb) potential surface presentation of ADDobody, Related to Figure 2. A front, side and back view (scale in kcal mol $^{-1}$ e $^{-1}$) show positively (blue) and negatively (red) charged patches. Interaction sites between ADDobodies forming a penton in the crystal are highlighted (blue circles). Loops (VL, RGD) are marked.

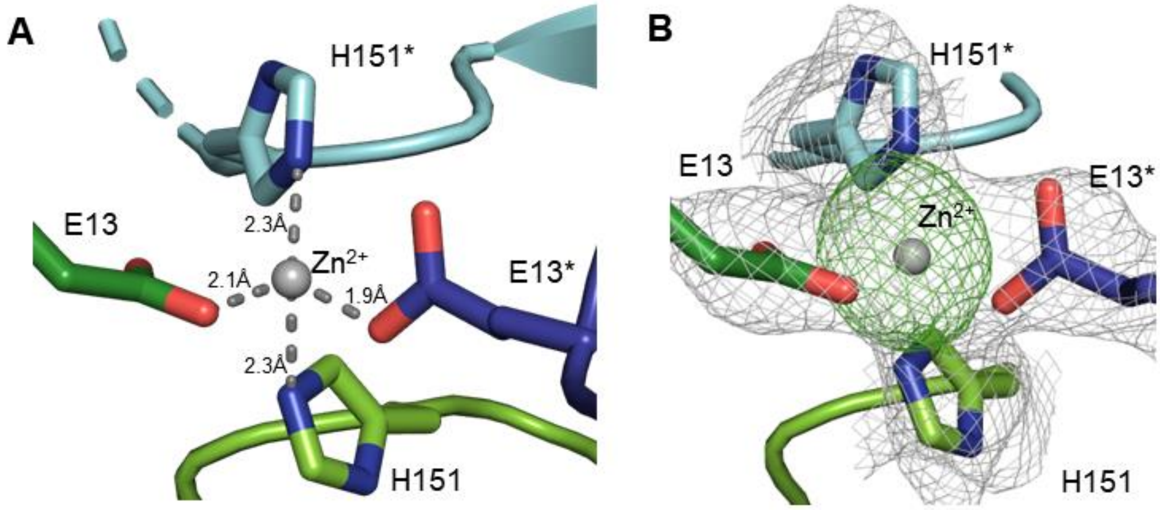

Figure S5: Binding of the coordinated zinc ion in the orthorhombic ADDobody crystal, Related to Figure 2. Zn^{2+} binding stabilizes the interaction between ADDobody penton rings in the crystal (see Fig. 2G). **(A)** A close-up view of the zinc ion coordination by histidine H151 and glutamate E13 residues from ADDobodies juxtaposed in the lattice. Residues of the same pentamer ring but different monomers are depicted in green shades; residues of the symmetry equivalent pentamer ring (marked with a star) are depicted in blue shades and distances to coordinating atoms are specified. **(B)** The $2mF_0\text{-}DF_c$ density is shown around the zinc ion and the coordinating residues as a grey mesh at 1σ, the *mFo-DFc* SA-omit map, calculated without Zinc ion, is depicted at 3σ as a green mesh.

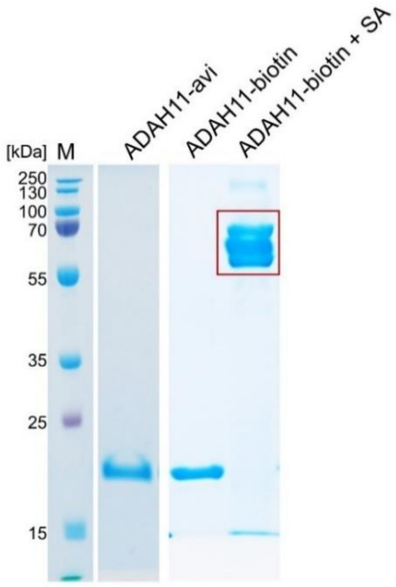

Figure S6: ADAH11-avi purification and biotinylation, Related to Figure 3. Left: Coomassie-stained SDS gel section showing purified ADAH11 with a C-terminal avi tag (Molecular weight: 21.7 kDa). Right: gel shift assay using biotinylated ADAH11 and tetrameric streptavidin (SA, 52 kDa). Bands of the biotinylated ADDobody-Streptavidin complex run at higher molecular weight (red box) indicating close to quantitative biotinylation. M: Molecular weight marker.

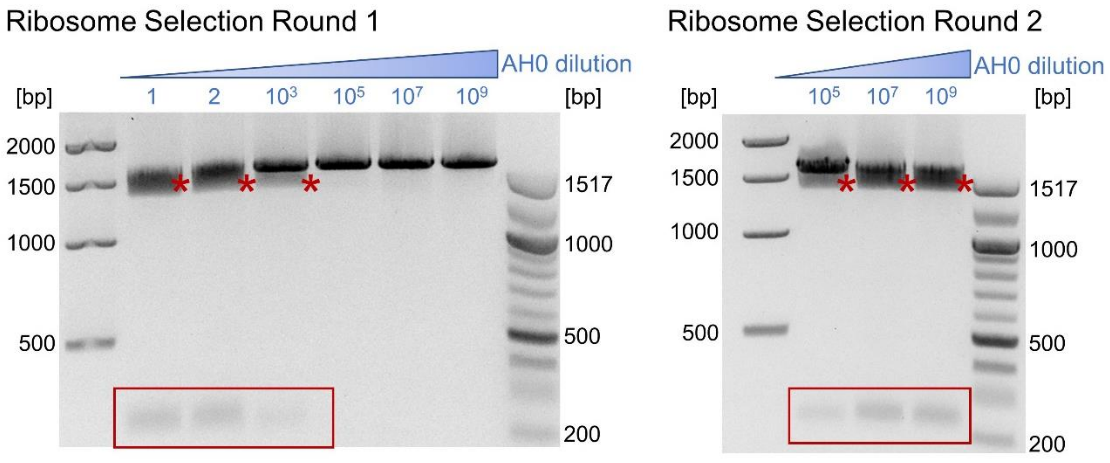

Figure S7. Proof-of-concept ribosome display selections, Related to Figure 3. DNA agarose gel analysis of *Pst*I restriction digestions of the PCR products obtained after 1 and 2 rounds of ribosome display selection starting from different dilutions of ADDobody-AH0 into ADDobody-57 using ADAH11 as the target antigen. Numbers in blue indicate x-fold dilution. Bands corresponding to the cut PCR product (encoding ADDobody-AH0) are highlighted by a star (1,408 bp) and a box (242 bp).

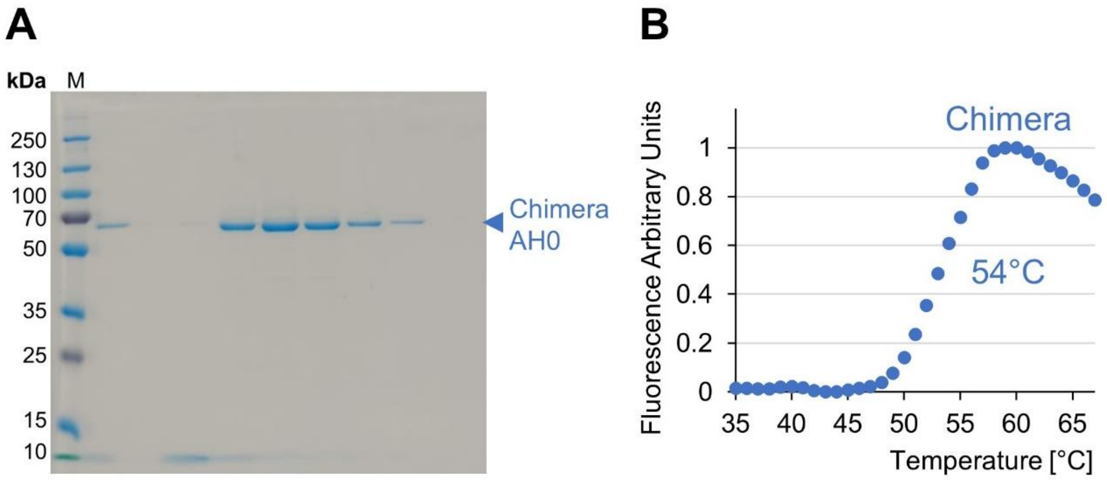

Figure S8. Chimera AH0 purification and thermostability, Related to Figure 4. **(A)** Coomassie-stained SDS gel showing purified Chimera AH0 after ion exchange chromatography (IEX). The peak fraction of eluted protein was used for electron microscopy (Fig. S10). **(B)** Thermal stability of Chimera was assessed by thermal unfolding measurements[27] exhibiting exceptional thermotolerance. A melting temperature (Tm) of 54ºC was obtained, which is virtually identical to the previously reported ADDomer Ad3 melting temperature[16].

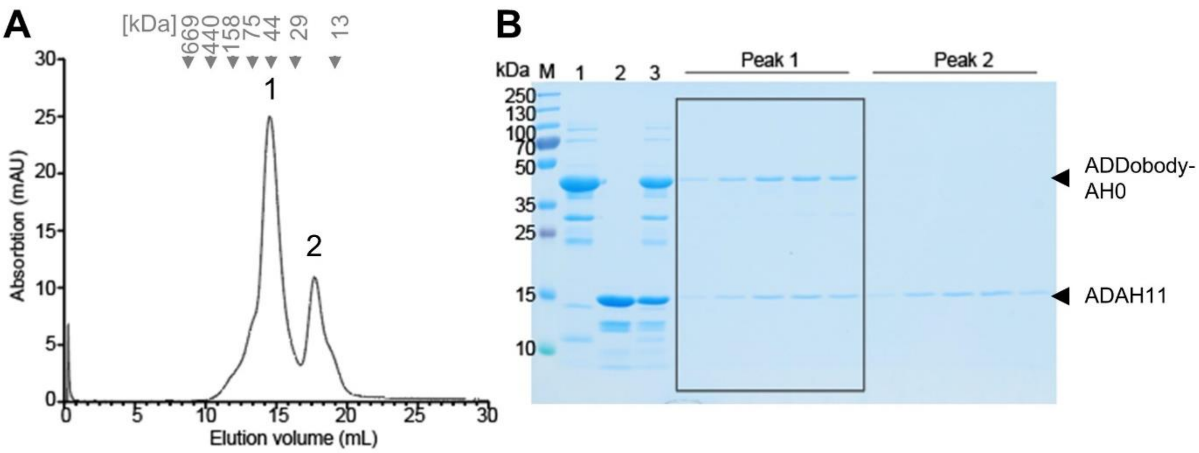

Figure S9. ADDobody-AH0 binding to ADAH11, Related to Figure 4. **(A)** Size-exclusion chromatography profile of a mix of ADDobody-AH0 and ADAH11 shows co-elution of the proteins, evidencing complex formation. ADDobody-AH0 and ADAH11 were mixed in a 1:1.5 molar ratio prior to loading onto a Superdex 200 10/300 GL column. **(B)** Coomassie-stained SDS gel showing the purified input samples ADDobody-AH0 (lane 1), ADAH11 target antigen (lane 2) and the mixture of the two proteins that was loaded on the column (lane 3). Fractions corresponding to Peak1 contain a complex between ADDobody-AH0 and ADAH11. Fractions corresponding to Peak2 contain unbound excess ADAH11. M: Molecular weight marker.

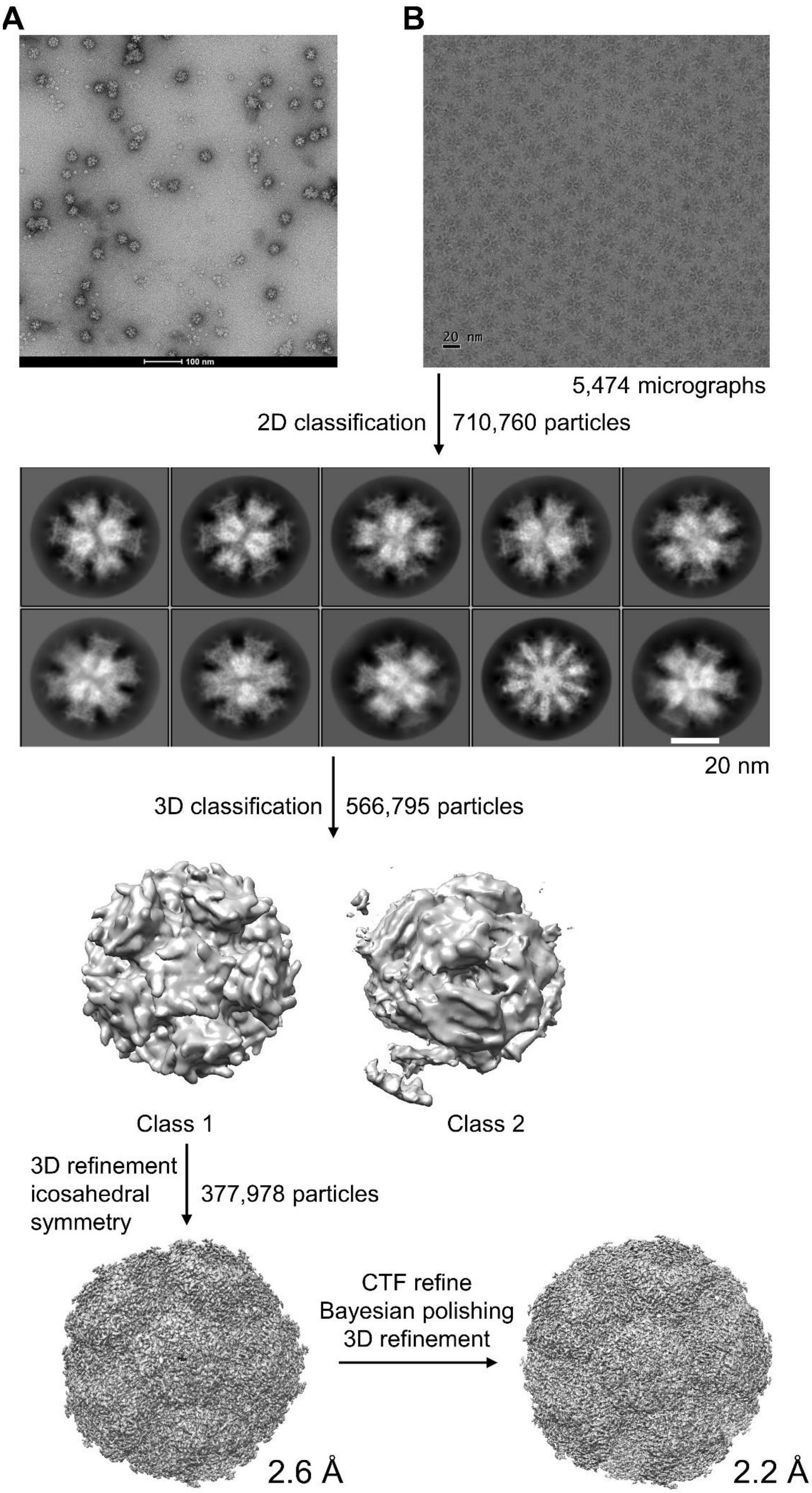
A
B
100 nm
20 nm
5,474 micrographs
2D classification
710,760 particles
20 nm
3D classification
566,795 particles
Class 1
Class 2
3D refinement
icosahedral
symmetry
377,978 particles
CTF refine
Bayesian polishing
3D refinement
2.6 Å
2.2 Å

Figure S10. Electron microscopy of Chimera AH0, Related to Figure 4. **(A)** A representative negative stain EM micrograph of Chimera AH0. Scale bar: 100nm **(B)** Cryo-EM image processing workflow. A motion-corrected cryo-EM micrograph (scale bar 20 nm), reference-free 2D class averages (scale bar 20 nm), 3D classification, application of icosahedral symmetry and 3D refinement resulted in a 2.2 Å cryo-EM map.

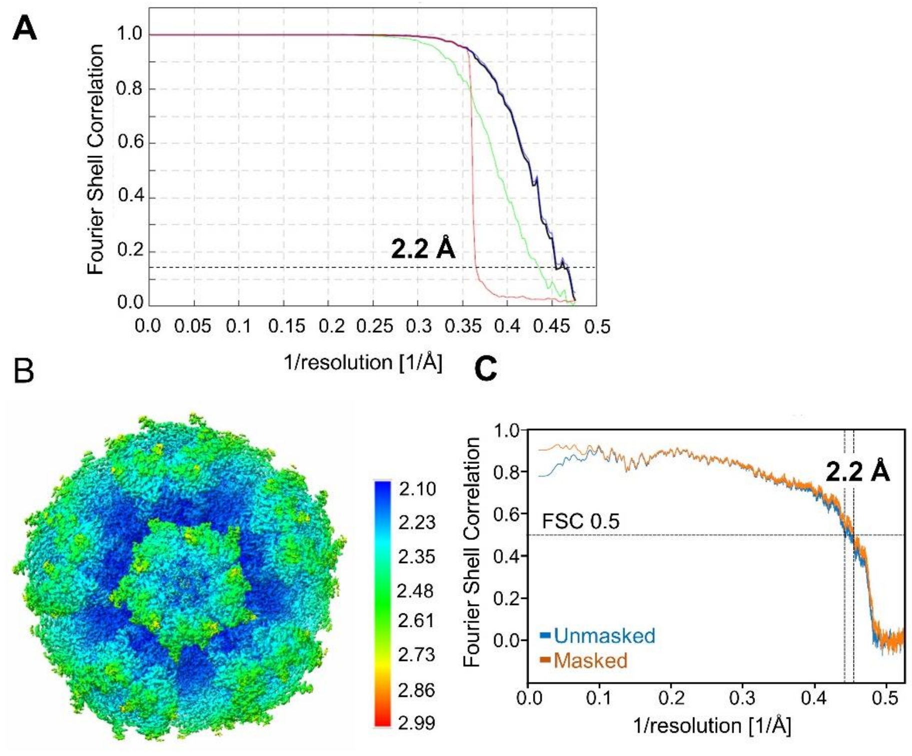

Figure S11. Chimera cryo-EM map and model resolution, Related to Figure 4. **(A)** The Fourier Shell correlation (FSC) curve after gold-standard refinement of 377,978 particles. The FSC = 0.143 criterion indicates an overall resolution of 2.2 Å. Blue curve: FSC curve of masked map, green curve: FSC curve of unmasked maps; red curve: FSC curve of phase randomized masked maps. **(B)** Local resolution of the final Chimera cryo-EM map calculated in RELION 3.1. The core of the complex is resolved at 2.2 Å whereas peripheral parts comprising the VL and RGD loops have a lower resolution of ~ 2.7 Å. **(C)** FSC curve calculated between the atomic model and the final cryo-EM map. The map/model FSC at 0.5 reaches a resolution of 2.2 Å.

Table S1. Cryo-EM data collection and refinement statistics, Chimera AH0, Related to Figure 4.

Voltage (kV)	200
Magnification (nominal)	130,000
Pixel size (Å/pix)	1.05 (0.525)
Flux (e-/pix/s)	5.9
Frames per exposure	40
Exposure (e-/ Å2)	1.06
Defocus range (µm)	-0.8 to -2.0
Micrographs collected	6179
Particles, final	377,978
Map sharpening B-factor (Å2)	-88
Masked resolution at 0.143 FSC (Å)	2.2

Refinement

Composition	
Amino acids	24180
RMSD bonds (Å)	0.004
RMSD angles (°)	0.747
Mean B-factor (Å)	
Amino acids	8.02
Ramachandran	
Favored (%)	94.30
Allowed (%)	5.70
Outliers (%)	0
Rotamer outlier (%)	0.64
Clash score	1.84
C-beta outliers (%)	0
CC (mask)	0.80
MolProbity score	1.33
EMRinger score	5.83
Model resolution (Å) 0.5 FSC threshold	2.2

Table S2. Ribosome display primers used in this study, Related to STAR Methods.

Primer	Sequence (5’ to 3’)
T7B_F_v3	5’ ATACGAAATTAATACGACTCACTATAGGGAGACCACAAC GGTTTCCCTCTAGAAATAATTTTG 3’
A1.MS-SDA-ADDobody-F	5’ AGACCACAACGGTTTCCCTCTAGAAATAATTTTGTTTAA CTTTAAGAAGGAGATATATATGGGATCCGGAATTCAACC 3’
tonBtot_R	5’CCGCACACCAGTAAGGTGTGCGGTCAGGATATTCACCA CAATCCC 3’
A14-tonB-ADDobody-R	5’ CCGCACACCAGTAAGGTGTGCGGTCAGGATATTCAC 3’
