# Supplementary material for: Engineering the ADDobody protein scaffold for generation of high-avidity ADDomer super-binders
Source: Structure. Author manuscript; Available in PMC 2024 Nov 15. (PMC7616808; doi:10.1016/j.str.2023.12.010)
Supplement: Supplemental information [file EMS199899-supplement-Supplemental_information.zip › 1-s2.0-S0969212623004513-mmc1.pdf]

Structure, Volume *32*

Supplemental Information

Engineering the ADDobody protein scaffold for generation of high-avidity ADDomer super-binders

Dora Buzas, Huan Sun, Christine Toelzer, Sathish K.N. Yadav, Ufuk Borucu, Gunjan Gautam, Kapil Gupta, Joshua C. Bufton, Julien Capin, Richard B. Sessions, Frederic Garzoni, Imre Berger, and Christiane Schaffitzel

Supplementary Information

Engineering the ADDobody protein scaffold for generation of high-avidity ADDomer super-binders

Dora Buzas[1,2,6], Huan Sun[1,2,3,6], Christine Toelzer[1], Sathish K. N. Yadav[1], Ufuk Borucu[1], Gunjan Gautam[1], Kapil Gupta[1,4], Joshua C. Bufton[1], Julien Capin[1], Richard B. Sessions[1], Frederic Garzoni[4], Imre Berger[1,2,5]* & Christiane Schaffitzel [1]*

[1] School of Biochemistry, University of Bristol, University Walk, Bristol BS8 1TD, UK

[2] Max Planck Bristol Centre for Minimal Biology, Cantock's Close, Bristol BS8 1TS, UK

[3] Present address: The Institute of Medicinal Plant Development (IMPLAD), No. 151 Malianwa North Road, Haidian District, Beijing 100193, P. R. China

[4] Imophoron Ltd, Science Creates Old Market, Midland Rd, Bristol BS2 0JZ, UK

[5] School of Chemistry, University of Bristol, Cantock's Close, Bristol BS8 1TS, UK

[6] These authors contributed equally.

* Correspondence: Christiane Schaffitzel, 0044 (0)117 394 1869; cb14941@bristol.ac.uk

Imre Berger, 0044 (0)117 394 1857; imre.berger@bristol.ac.uk

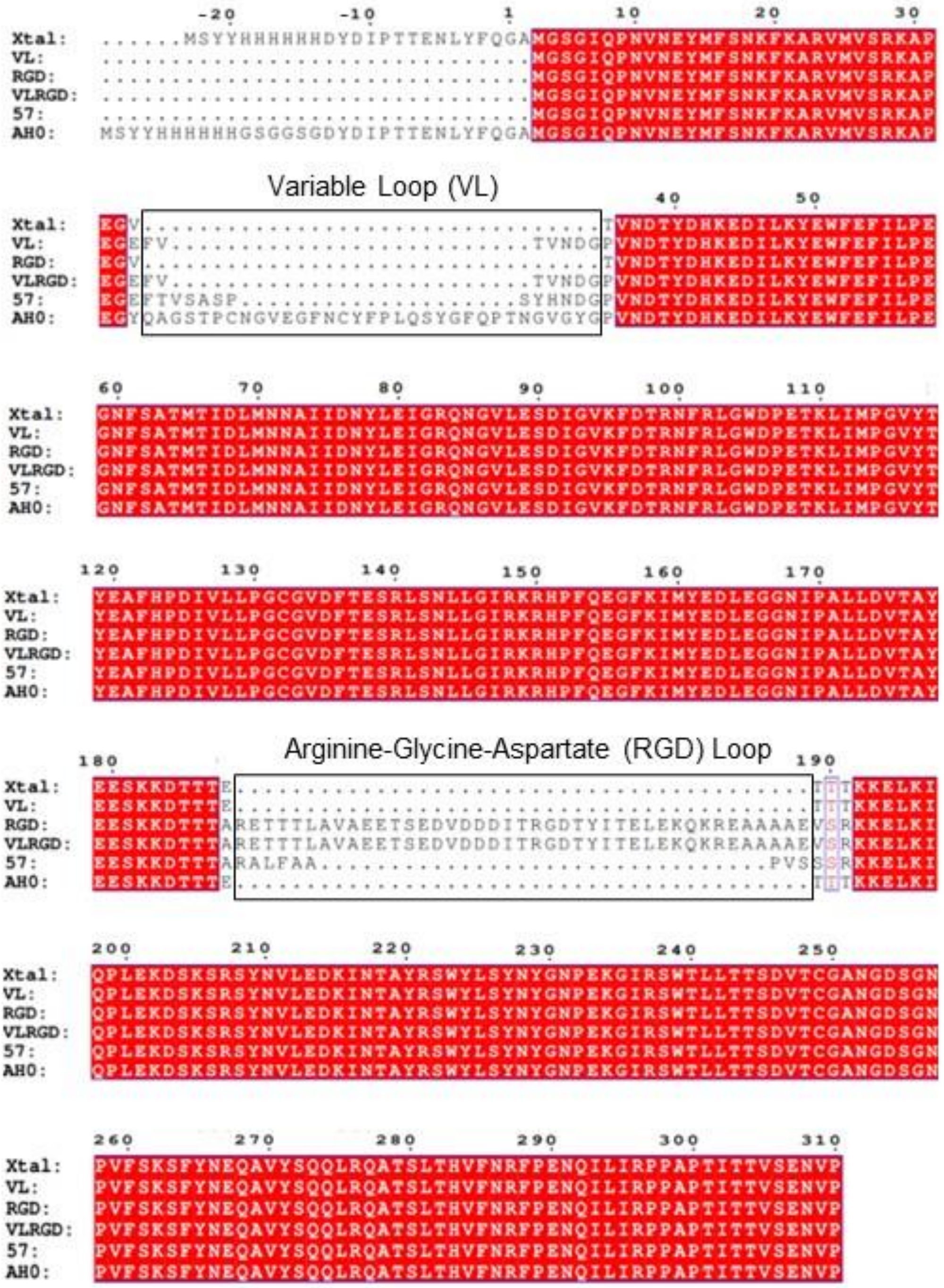

Figure S1: Amino-acid sequences of ADDobodies used in this study, Related to Figure 1, 2 and 3.

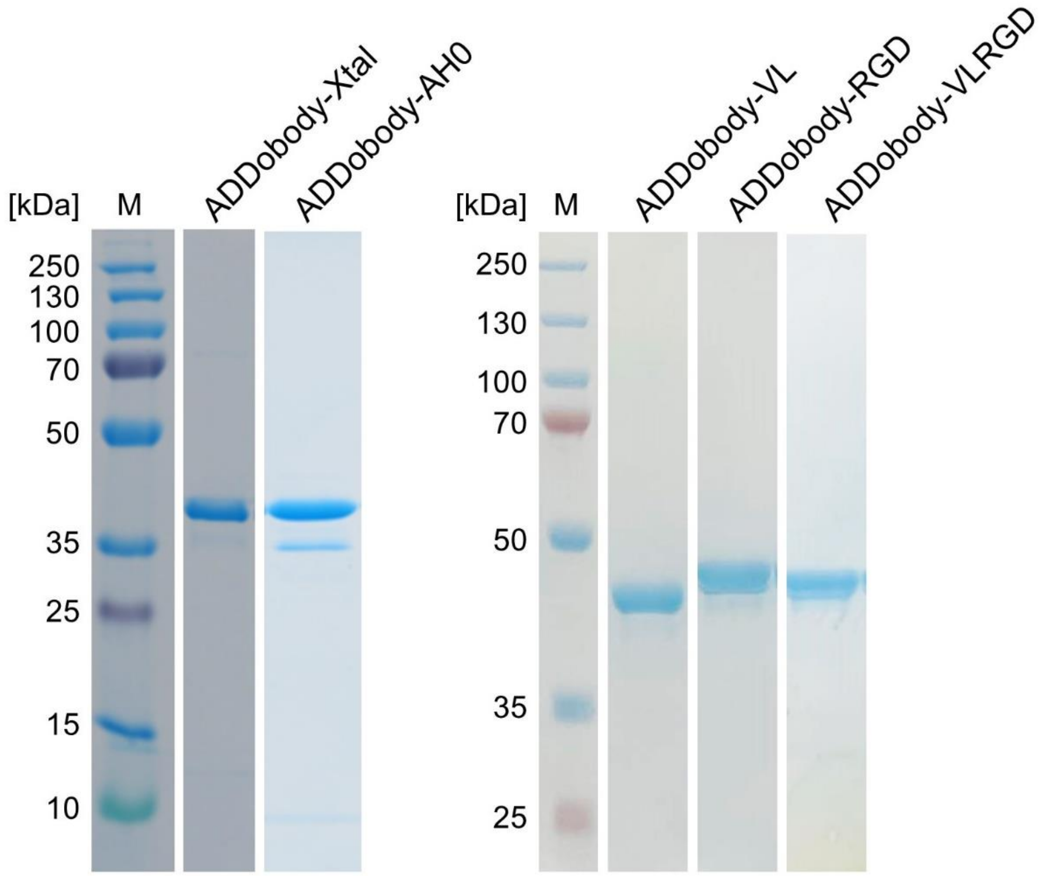

Figure S2. Coomassie-stained SDS gel showing purified ADDobodies, Related to Figure 1. Coomassie-stained SDS–PAGE showing purified ADDobody constructs. M: Molecular weight marker. The sequence of the ADDobodies is provided in Fig. S1.

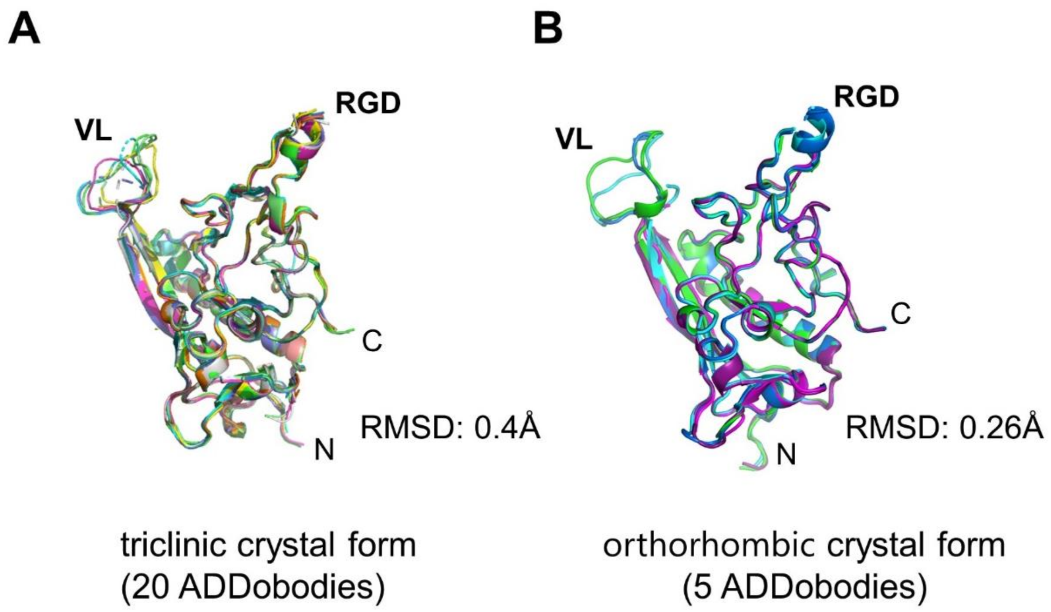

Figure S3. Superimposition of ADDobodies, Related to Figure 2. **(A)** from the triclinic (P1) crystal form comprising 20 ADDobody molecules in the asymmetric unit, adopting four pentons forming two decamer barrels, and **(B)** from the Zn^{2+}-containing orthorhombic ($P2_12_12_1$) crystal form (5 ADDobodies per asymmetric unit adopting a penton). The root-mean-square deviation (RMSD) of the overlay is indicated.

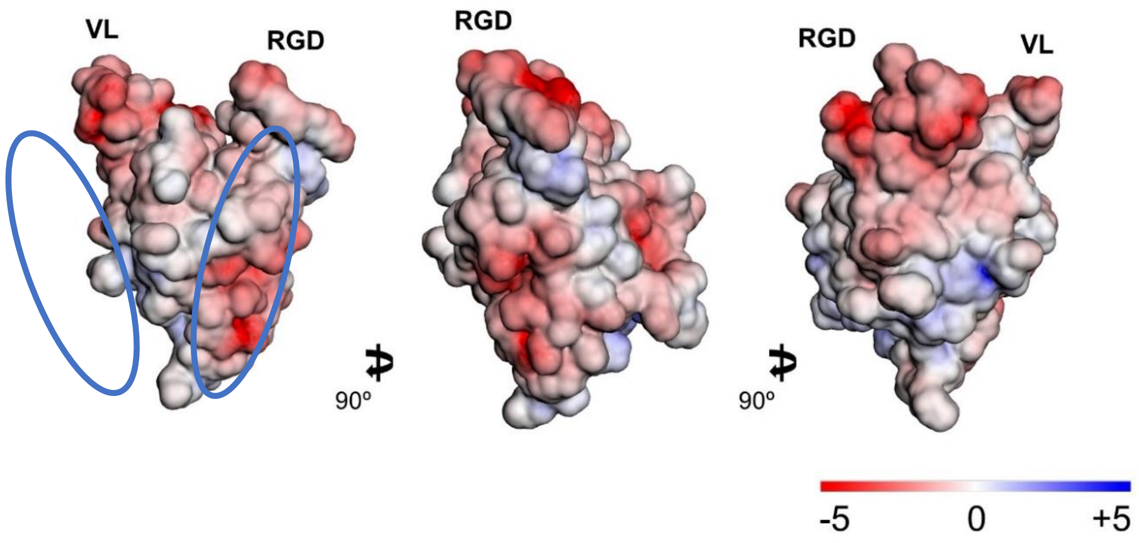

Figure S4. Electrostatic (Coulomb) potential surface presentation of ADDobody, Related to Figure 2. A front, side and back view (scale in kcal mol^{-1} e^{-1}) show positively (blue) and negatively (red) charged patches. Interaction sites between ADDobodies forming a penton in the crystal are highlighted (blue circles). Loops (VL, RGD) are marked.

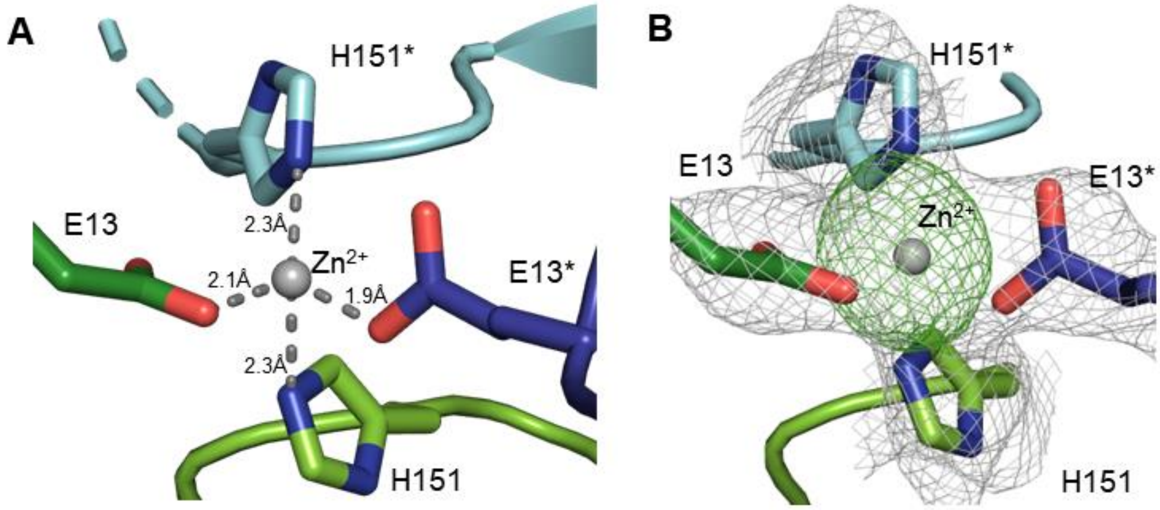

Figure S5: Binding of the coordinated zinc ion in the orthorhombic ADDobody crystal, Related to Figure 2. Zn^{2+} binding stabilizes the interaction between ADDobody penton rings in the crystal (see Fig. 2G). **(A)** A close-up view of the zinc ion coordination by histidine H151 and glutamate E13 residues from ADDobodies juxtaposed in the lattice. Residues of the same pentamer ring but different monomers are depicted in green shades; residues of the symmetry equivalent pentamer ring (marked with a star) are depicted in blue shades and distances to coordinating atoms are specified. **(B)** The $2mF_0\text{-}DF_c$ density is shown around the zinc ion and the coordinating residues as a grey mesh at 1σ, the *mFo-DFc* SA-omit map, calculated without Zinc ion, is depicted at 3σ as a green mesh.

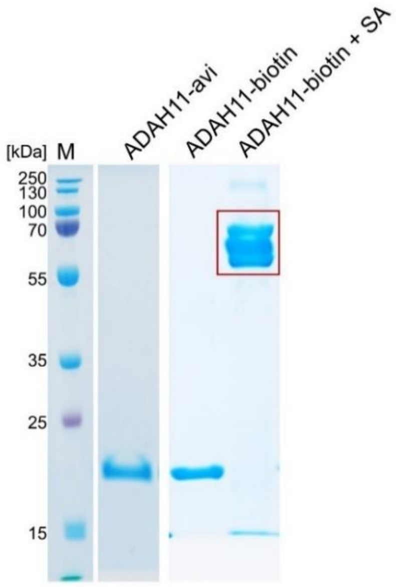

Figure S6: ADAH11-avi purification and biotinylation, Related to Figure 3. Left: Coomassie-stained SDS gel section showing purified ADAH11 with a C-terminal avi tag (Molecular weight: 21.7 kDa). Right: gel shift assay using biotinylated ADAH11 and tetrameric streptavidin (SA, 52 kDa). Bands of the biotinylated ADDobody-Streptavidin complex run at higher molecular weight (red box) indicating close to quantitative biotinylation. M: Molecular weight marker.

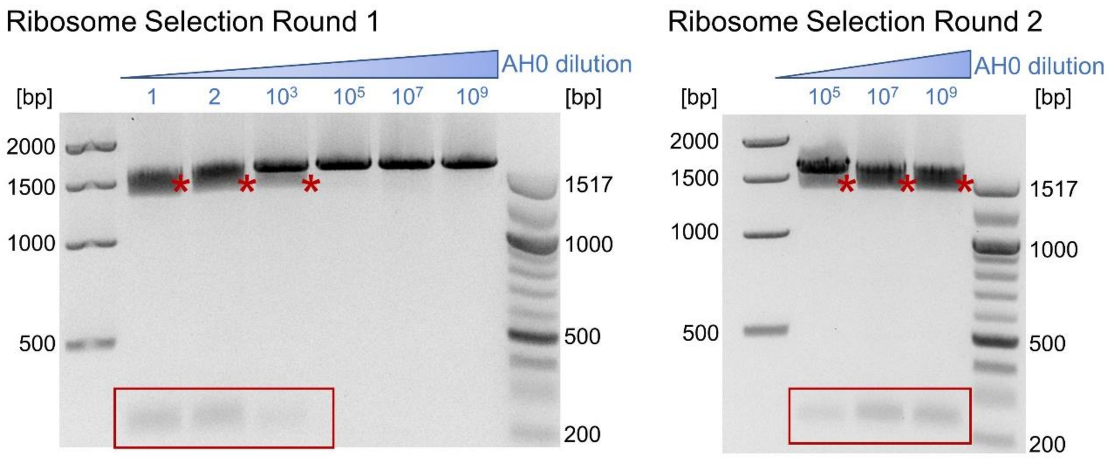

Figure S7. Proof-of-concept ribosome display selections, Related to Figure 3. DNA agarose gel analysis of *Pst*I restriction digestions of the PCR products obtained after 1 and 2 rounds of ribosome display selection starting from different dilutions of ADDobody-AH0 into ADDobody-57 using ADAH11 as the target antigen. Numbers in blue indicate x-fold dilution. Bands corresponding to the cut PCR product (encoding ADDobody-AH0) are highlighted by a star (1,408 bp) and a box (242 bp).

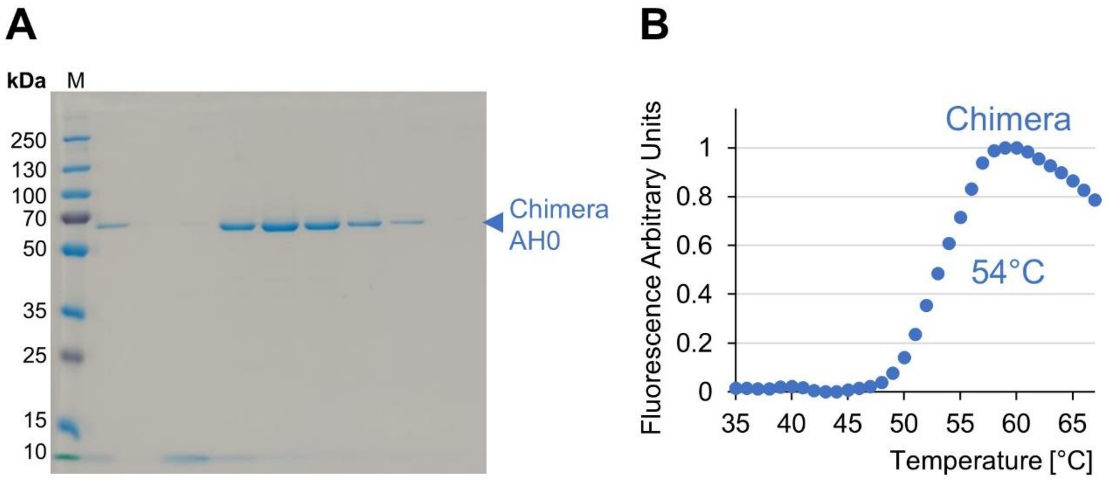

Figure S8. Chimera AH0 purification and thermostability, Related to Figure 4. **(A)** Coomassie-stained SDS gel showing purified Chimera AH0 after ion exchange chromatography (IEX). The peak fraction of eluted protein was used for electron microscopy (Fig. S10). **(B)** Thermal stability of Chimera was assessed by thermal unfolding measurements[27] exhibiting exceptional thermotolerance. A melting temperature (Tm) of 54ºC was obtained, which is virtually identical to the previously reported ADDomer Ad3 melting temperature[16].

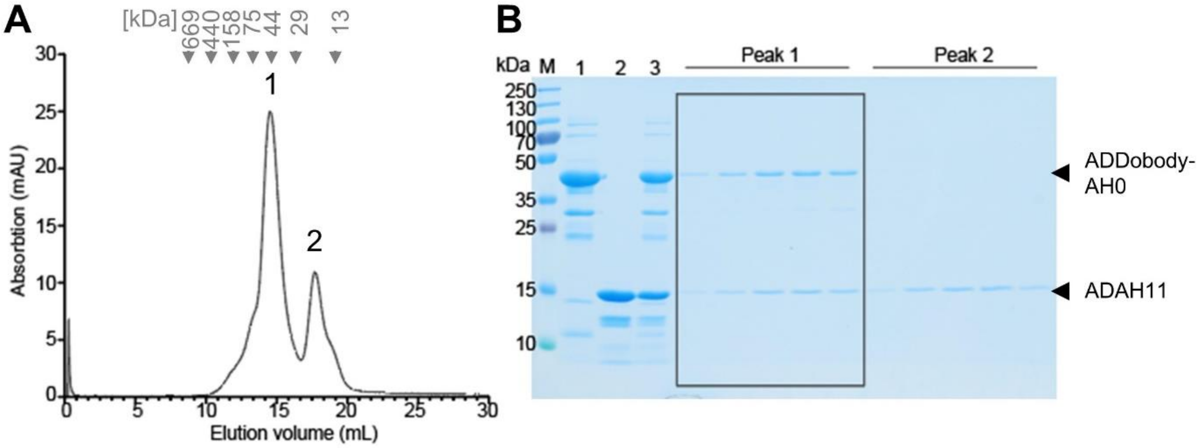

Figure S9. ADDobody-AH0 binding to ADAH11, Related to Figure 4. **(A)** Size-exclusion chromatography profile of a mix of ADDobody-AH0 and ADAH11 shows co-elution of the proteins, evidencing complex formation. ADDobody-AH0 and ADAH11 were mixed in a 1:1.5 molar ratio prior to loading onto a Superdex 200 10/300 GL column. **(B)** Coomassie-stained SDS gel showing the purified input samples ADDobody-AH0 (lane 1), ADAH11 target antigen (lane 2) and the mixture of the two proteins that was loaded on the column (lane 3). Fractions corresponding to Peak1 contain a complex between ADDobody-AH0 and ADAH11. Fractions corresponding to Peak2 contain unbound excess ADAH11. M: Molecular weight marker.

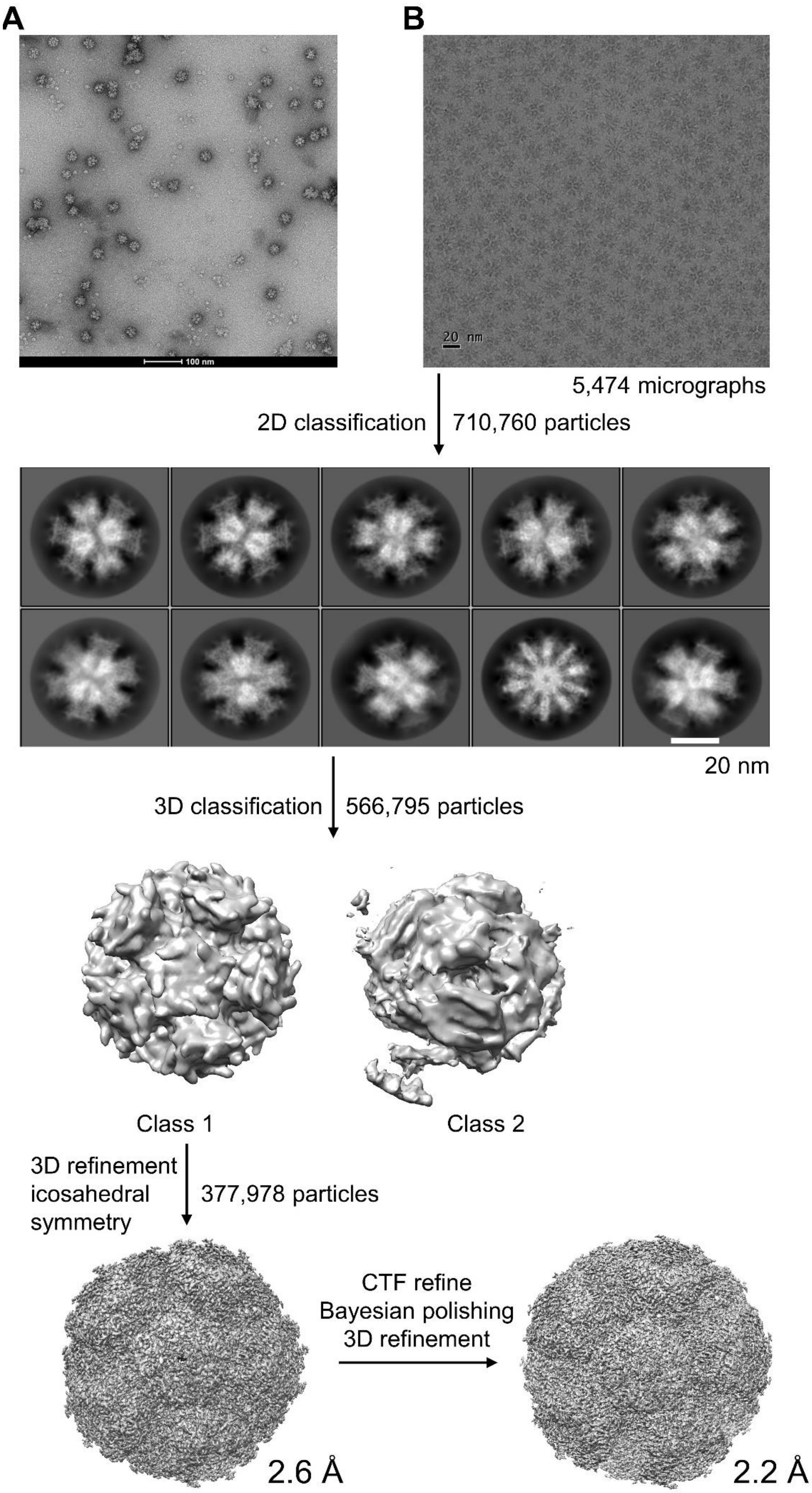
A
B
100 nm
20 nm
5,474 micrographs
2D classification
710,760 particles
20 nm
3D classification
566,795 particles
Class 1
Class 2
3D refinement
icosahedral
symmetry
377,978 particles
CTF refine
Bayesian polishing
3D refinement
2.6 Å
2.2 Å

Figure S10. Electron microscopy of Chimera AH0, Related to Figure 4. **(A)** A representative negative stain EM micrograph of Chimera AH0. Scale bar: 100nm **(B)** Cryo-EM image processing workflow. A motion-corrected cryo-EM micrograph (scale bar 20 nm), reference-free 2D class averages (scale bar 20 nm), 3D classification, application of icosahedral symmetry and 3D refinement resulted in a 2.2 Å cryo-EM map.

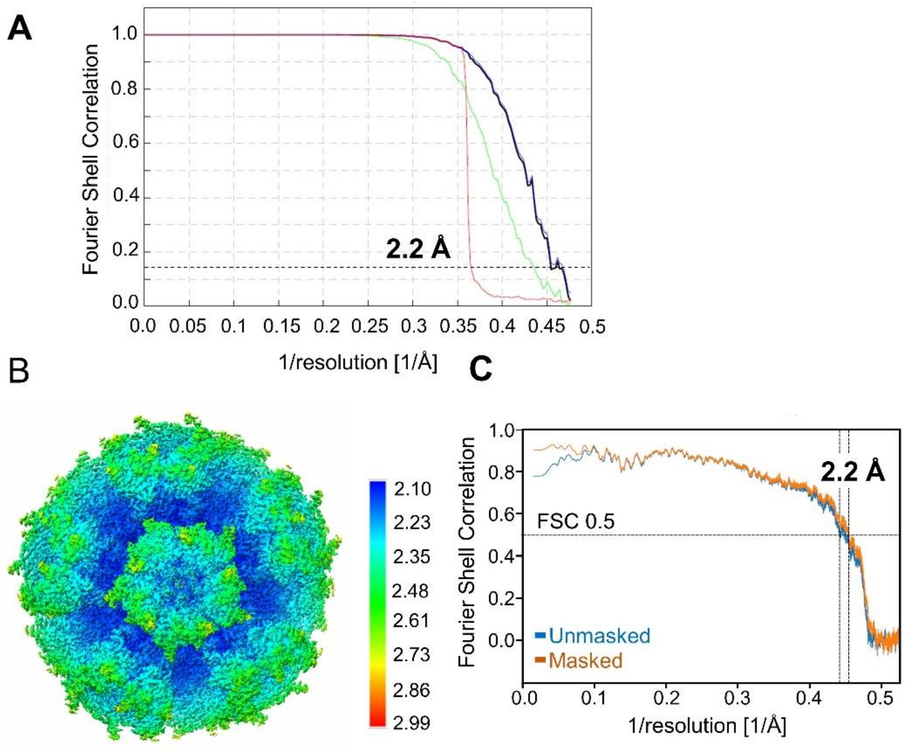

Figure S11. Chimera cryo-EM map and model resolution, Related to Figure 4. **(A)** The Fourier Shell correlation (FSC) curve after gold-standard refinement of 377,978 particles. The FSC = 0.143 criterion indicates an overall resolution of 2.2 Å. Blue curve: FSC curve of masked map, green curve: FSC curve of unmasked maps; red curve: FSC curve of phase randomized masked maps. **(B)** Local resolution of the final Chimera cryo-EM map calculated in RELION 3.1. The core of the complex is resolved at 2.2 Å whereas peripheral parts comprising the VL and RGD loops have a lower resolution of ~ 2.7 Å. **(C)** FSC curve calculated between the atomic model and the final cryo-EM map. The map/model FSC at 0.5 reaches a resolution of 2.2 Å.

Table S1. Cryo-EM data collection and refinement statistics, Chimera AH0, Related to Figure 4.

Voltage (kV)	200
Magnification (nominal)	130,000
Pixel size (Å/pix)	1.05 (0.525)
Flux (e-/pix/s)	5.9
Frames per exposure	40
Exposure (e-/ Å2)	1.06
Defocus range (µm)	-0.8 to -2.0
Micrographs collected	6179
Particles, final	377,978
Map sharpening B-factor (Å2)	-88
Masked resolution at 0.143 FSC (Å)	2.2

Refinement

Composition	
Amino acids	24180
RMSD bonds (Å)	0.004
RMSD angles (°)	0.747
Mean B-factor (Å)	
Amino acids	8.02
Ramachandran	
Favored (%)	94.30
Allowed (%)	5.70
Outliers (%)	0
Rotamer outlier (%)	0.64
Clash score	1.84
C-beta outliers (%)	0
CC (mask)	0.80
MolProbity score	1.33
EMRinger score	5.83
Model resolution (Å) 0.5 FSC threshold	2.2

Table S2. Ribosome display primers used in this study, Related to STAR Methods.

Primer	Sequence (5' to 3')
T7B_F_v3	5' ATACGAAATTAATACGACTCACTATAGGGAGACCACAAC GGTTTCCCTCTAGAAATAATTTTG 3'
A1.MS-SDA-ADDobody-F	5' AGACCACAACGGTTTCCCTCTAGAAATAATTTTGTTTAA CTTTAAGAAGGAGATATATATGGGATCCGGAATTCAACC 3'
tonBtot_R	5'CCGCACACCAGTAAGGTGTGCGGTCAGGATATTCACCA CAATCCC 3'
A14-tonB-ADDobody-R	5' CCGCACACCAGTAAGGTGTGCGGTCAGGATATTCAC 3'
